# miRNA–target chimeras reveal miRNA 3′-end pairing as a major determinant of Argonaute target specificity

**DOI:** 10.1038/ncomms9864

**Published:** 2015-11-25

**Authors:** Michael J. Moore, Troels K. H. Scheel, Joseph M. Luna, Christopher Y. Park, John J. Fak, Eiko Nishiuchi, Charles M. Rice, Robert B. Darnell

**Affiliations:** 1 Laboratory of Molecular Neuro-Oncology and Howard Hughes Medical Institute, The Rockefeller University, 1230 York Avenue, Box 226, New York, New York 10065, USA; 2 Laboratory of Virology and Infectious Disease, Center for the Study of Hepatitis C, The Rockefeller University, New York, New York 10065, USA; 3 Copenhagen Hepatitis C Program (CO-HEP), Department of Infectious Diseases and Clinical Research Centre, Copenhagen University Hospital, 2650 Hvidovre, Denmark; 4 Department of Immunology and Microbiology, Faculty of Health and Medical Sciences, University of Copenhagen, 2200 Copenhagen, Denmark; 5 New York Genome Center, 101 Avenue of the Americas, New York, New York 10013, USA

## Abstract

microRNAs (miRNAs) act as sequence-specific guides for Argonaute (AGO) proteins, which mediate posttranscriptional silencing of target messenger RNAs. Despite their importance in many biological processes, rules governing AGO–miRNA targeting are only partially understood. Here we report a modified AGO HITS-CLIP strategy termed CLEAR (covalent ligation of endogenous Argonaute-bound RNAs)-CLIP, which enriches miRNAs ligated to their endogenous mRNA targets. CLEAR-CLIP mapped ∼130,000 endogenous miRNA–target interactions in mouse brain and ∼40,000 in human hepatoma cells. Motif and structural analysis define expanded pairing rules for over 200 mammalian miRNAs. Most interactions combine seed-based pairing with distinct, miRNA-specific patterns of auxiliary pairing. At some regulatory sites, this specificity confers distinct silencing functions to miRNA family members with shared seed sequences but divergent 3′-ends. This work provides a means for explicit biochemical identification of miRNA sites *in vivo*, leading to the discovery that miRNA 3′-end pairing is a general determinant of AGO binding specificity.

## Abstract (web summary)

microRNAs (miRNAs) act as sequence-specific guides for Argonaute (AGO) proteins. By using a modified AGO HITS-CLIP strategy that enriches miRNAs ligated to their endogenous mRNA targets, here the authors show that miRNA 3' end pairing is a general determinant of AGO binding specificity.

## Graphical abstract

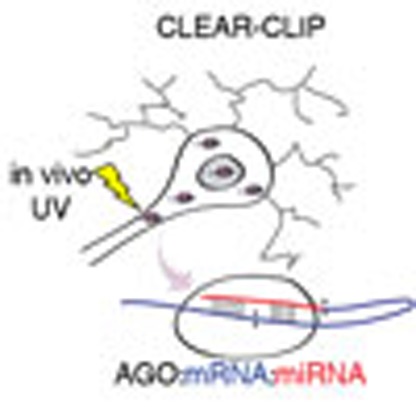

microRNAs (miRNAs) are small, non-coding RNAs that mediate posttranscriptional RNA silencing by sequence-specific targeting of Argonaute (AGO) proteins to mRNAs^1^. miRNAs regulate the development, homeostasis and pathologies of virtually all vertebrate tissues. Many miRNAs have specific or enriched expression in the central nervous system, regulating such diverse processes as neuronal differentiation, excitation, synaptogenesis and plasticity^2^. Accordingly, miRNA dysregulation is implicated in neurological disorders and many cancers including glioma and liver cancer^3^,^4^,^5^. However, miRNA function in these contexts remains unclear, as most *in vivo* mRNA targets are unknown.

Accurate miRNA target identification remains a formidable challenge^6^. Canonical miRNA binding involves base pairing of the miRNA seed region (nucleotides 2–8) to complementary target sites^7^,^8^. Such short motifs occur frequently in the transcriptome and are not sufficient to predict miRNA binding, leading to high false discovery rates for purely bioinformatic predictions^9^. To mitigate this limitation, evolutionary conservation and local AU sequence content are employed as screens for site functionality and accessibility, respectively^7^,^10^. However, the importance of non-conserved miRNA regulation, especially in the brain^11^, and limitations of context predictions without empirical binding information are well established^12^. Moreover, the assumption of uniform rules for all miRNAs ignores non-canonical miRNA binding, increasingly recognized as widespread^13^,^14^,^15^. Rules beyond seed-based pairing such as supplementary pairing of miRNA 3′-bases 12–17 have been described but are generally considered rare^16^,^17^,^18^. Other non-canonical binding modes include 3′-end centric ‘seedless' pairing^19^,^20^, centred miRNA pairing^21^ and nucleation bulges in the seed region^13^.

Empirical mapping of miRNA target sites *in vivo* was first achieved with ultraviolet cross-linking and immunoprecipitation with high-throughput sequencing (HITS-CLIP) of AGO proteins^22^,^23^,^24^. AGO HITS-CLIP generates two data sets—a transcriptome-wide target binding map and an empirical catalogue of expressed miRNAs—that empower accurate identification of functional miRNA-binding sites. However, the inability to link miRNA and target unambiguously remains a limitation. Two groups reported experimental strategies to ligate miRNA to target RNA in purified AGO complexes. CLASH (cross-linking and sequencing of hybrids) identified thousands of miRNA–target chimeras using dual-tagged AGO1 in HEK-293T cells, revealing frequent seed-independent miRNA binding^19^,^25^. Soon after, modified photoactivatable ribonucleoside-enhanced CLIP identified ∼3,600 unambiguous events in *Caenorhabditis elegans*^26^. Although identifying thousands of novel interactions, the reliance of these studies on exogenous AGO expression excludes them from analysis of human tissues and, currently, *in vivo* mouse models, and raises concerns about the stoichiometry of RNA-binding events.

We have developed modifications of AGO HITS-CLIP, termed CLEAR (covalent ligation of endogenous Argonaute-bound RNAs)-CLIP, permitting isolation of miRNA–target chimeras from endogenous AGO–miRNA–mRNA complexes. CLEAR-CLIP identifies tens of thousands of miRNA target sites in mouse brain including novel targets for many neuron-specific miRNAs. In mouse brain and human liver cells, we define expanded pairing rules for over 200 mammalian miRNAs illustrating widespread use of miRNA 3′-end auxiliary pairing *in vivo* and tolerance of diverse, although constrained, pairing patterns for many miRNAs. Integrated with HITS-CLIP binding information, CLEAR-CLIP provides an improved empirical basis for identification of physiologic canonical and non-canonical miRNA regulation.

## Results

### CLEAR-CLIP defines miRNA–target interactions *in vivo*

**Figure 1:**
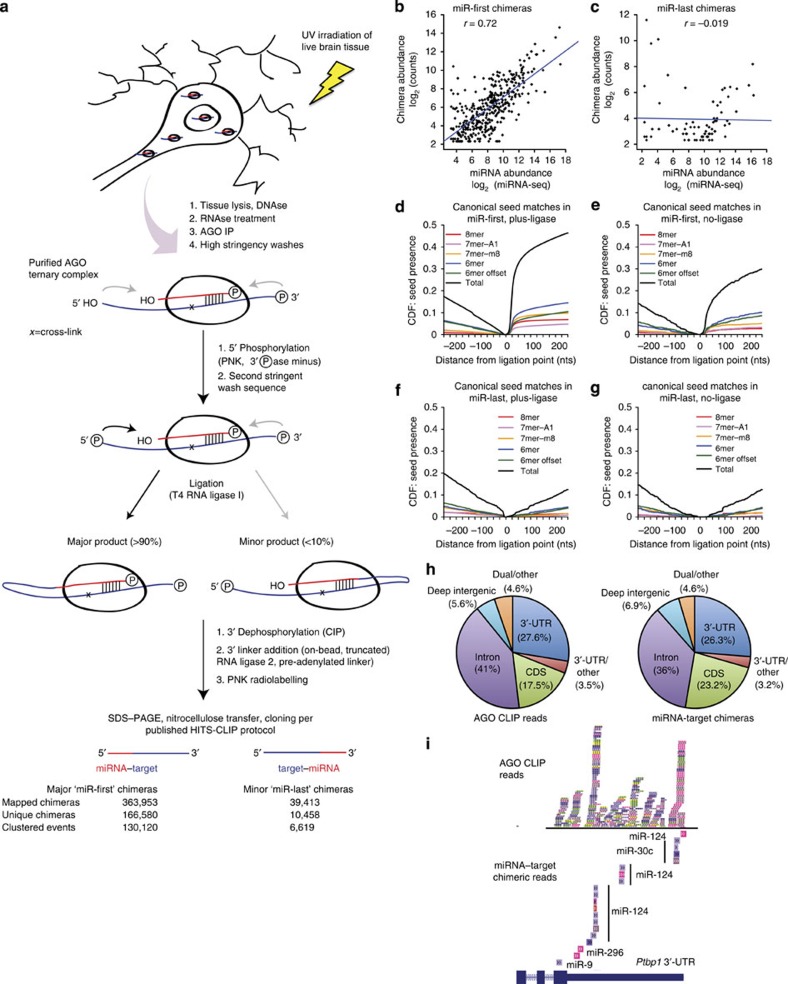
CLEAR-CLIP unambiguously identifies endogenous *in vivo* miRNA–target interactions. (**a**) In CLEAR-CLIP, AGO–target contacts are cross-linked *in vivo* by ultraviolet irradiation. Endogenous AGO is immunopurified from tissue lysates and washed under stringent conditions that disrupt the interaction of AGO–miRNA with non-cross-linked target RNAs. Target regions cannot be cloned from no-ultraviolet controls, indicating that cross-linking of AGO to target mRNA (shown as ‘X') is required. Cross-linking of the miRNA may not be necessary, because the AGO–miRNA interaction is uniquely strong and survives stringent washing. After washing, RNA ends are modified to facilitate miRNA–target ligation and joined with T4 RNA Ligase I treatment, yielding miRNA–target chimeric RNAs in two orientations at the indicated frequencies. All depicted post-IP manipulations up to SDS–PAGE occur on beads. Correlation plots of miRNA abundance of all miR-first (**b**) and miR-last (**c**) chimeras versus small RNA sequencing data in the brain^67^. Pearson's correlation coefficients (*r*) are shown. CDF plots of cognate miRNA seed matches in target regions relative to ligation site for all miR-first chimeras in plus-ligase (**d**) and no-ligase (**e**) samples, and for all miR-last chimeras in plus-ligase (**f**) and no-ligase (**g**) samples. (**h**) Distribution of standard AGO CLIP and miRNA–target chimeras in transcript regions. (**i**) CLEAR-CLIP confirmed known miRNA regulation, here exemplified by miR-124 regulation of the *Ptbp1* 3′-UTR. Other examples are shown in Supplementary Fig. 3c.

We modified AGO HITS-CLIP to facilitate direct ligation of miRNA and target RNA. Endogenous AGO–RNA complexes were purified from ultraviolet-irradiated mouse brain neocortex using monoclonal anti-AGO and were washed in stringent conditions that disrupt native AGO–mRNA interactions (Fig. 1a)^22^,^27^. Complexes were treated with dilute RNAse to generate footprint-sized fragments. To test whether T4 RNA ligase I treatment could join free RNA ends, AGO–RNA was radiolabelled with polynucleotide kinase (PNK) and ^32^P-γ-ATP, then treated with RNA ligase. Complexes were treated with alkaline phosphatase and visualized by SDS–polyacrylamide gel electrophoresis (PAGE) and autoradiography to assess dephosphorylation. Compared with untreated samples, ligase-treated complexes were ‘protected' from dephosphorylation, indicating ligation of RNA ends (Supplementary Fig. 1a). Using optimized ligation conditions, 12 biological replicates from post-natal day 13 (P13)-aged mouse neocortex were prepared, along with two no-ligase control samples omitting RNA ligase I treatment. Pre-adenylated 3′-adapter was added on-bead with truncated RNA ligase 2, which cannot catalyse standard RNA–RNA ligation^28^. Isolation, cloning and sequencing of AGO-bound RNA tags retrieved hundreds of thousands of miRNA–target chimeric reads in addition to standard target and miRNA fragments (Supplementary Table 1). We termed this method CLEAR-CLIP.

CLEAR-CLIP yielded miRNA–target chimeras in two orientations, termed miR-first and miR-last based on the position of miRNA and target fragments (Fig. 1a). Most chimeras contained full-length miRNAs and miR-first chimeras were on-average 14-fold more frequent than miR-last. Uniquely mapped miR-first chimeras were ∼1.5–5% of total unique reads in ligase-treated samples, but only ∼0.2–0.3% in no-ligase samples. miR-last chimeras were ∼0.05–0.2% of unique reads, irrespective of ligase treatment. Thus, most miR-first chimeras were dependent on exogenous ligase but miR-last chimeras were not. Importantly, chimeric and non-chimeric mRNA target sequences could not be cloned from no-ultraviolet controls, indicating that *in vivo* AGO–mRNA ultraviolet cross-linking was strictly required for CLEAR-CLIP.

miRNA frequency in miR-first chimeras correlated with brain miRNA abundance (Fig. 1b and Supplementary Fig. 1b–d). miR-first chimeras were dominated by a small number brain-abundant miRNAs (Supplementary Fig. 1e). In contrast, miR-last chimeras did not correlate to miRNA abundance and were dominated by dubiously annotated miRNAs (Fig. 1c and Supplementary Fig. 1d). Target regions in miR-first chimeras were also strongly enriched for canonical seed matches to their cognate miRNAs (Fig. 1d). Seed enrichment occurred within ∼75 nt of the miRNA ligation junction in the expected downstream (3′) region, but not the upstream region (5′) (Fig. 1d). Consistent with prior findings, chimeras were present at low levels in no-ligase samples^26^, although with reduced seed enrichments (Fig. 1e). For miR-last chimeras, the reversed pattern of seed distribution around the ligation junction was expected; however, this pattern was weak in ligase-treated samples and was absent in no-ligase samples (Fig. 1f,g). As they better reflected miRNA abundance and known miRNA targeting features, we focused exclusively on miR-first chimeras (henceforth ‘chimeras').

Notably, many CLEAR-CLIP target regions lacked canonical seed matches (Fig. 1d), consistent with similar analyses^19^,^26^. We took two approaches to assess miRNA ligation to non-cross-linked targets, which could falsely identify non-physiologic interactions. First, we tested chimera ligation after denaturing AGO complexes in 6M guanidine hydrochloride, as in CLASH^19^. Interactions from denatured samples were similar to other samples based on miRNA seed match frequency, indicating *bona fide* interactions. However, compared with other samples, the yield of chimeric and non-chimeric CLIP reads was low (Supplementary Table 1) and skewed to non-genic sites (Supplementary Fig. 1f); thus, we pursued it no further.

Second, we performed mixing experiments to assess miRNA ligation to non-target sequences after postlysis re-association. CLEAR-CLIP was done on lysates from cross-linked mouse cortex mixed with *Escherichia coli* total RNA, which contains thousands of potential miRNA sites by random chance at a per-nucleotide frequency comparable to mouse. For two replicates each, equal mass amounts of mouse and *E. coli* RNA or a large excess of *E. coli* RNA (sixfold) were mixed. We confirmed that *E. coli* RNA was not degraded in brain lysates (Supplementary Fig. 2). Across four mouse-only control samples, 1% of chimeric CLIP reads mapped to the *E. coli* genome, establishing the ‘background' from cross-mapped reads and minute RNA contaminants from commercial enzymes^29^ (Supplementary Table 2). Average *E. coli* mapping rates were 1.9% in equal-mixture samples and 5.2% in excess-mixture samples. To examine a more complex competitor RNA pool, we performed CLEAR-CLIP on mixed lysates from ultraviolet-irradiated mouse brain and non-cross-linked *Drosophila* S2 cells containing equal amounts of RNA. Here, 0.7% of mouse-only chimeric sequences mapped to the *Drosophila* genome compared with 2.9% of mixed mouse/fly samples (Supplementary Table 2). Collectively, these experiments indicate low (<5%) false discovery comparable to related methods^19^.

### CLEAR-CLIP enhances the brain miRNA regulatory map

Chimeras with the same miRNA and overlapping genomic coordinates were clustered to yield 130,120 brain miRNA–target interactions (Fig. 1a and Supplementary Data 1). Seventy-nine per cent (102,882) of interactions were also supported by non-chimeric AGO CLIP reads. We combined chimeric CLEAR-CLIP reads with conventional CLIP reads from 15 total biological replicates, to generate an enhanced brain miRNA regulatory map. We identified 96,685 AGO peaks supported in at least 5 mice, defined as biological complexity (BC)≥5 (Supplementary Data 2)^22^. Twenty-seven per cent of BC≥5 peaks (26,304) had chimera support unambiguously identifying the miRNA(s) and this proportion increased substantially for peaks with greater BC (Supplementary Fig. 1g). Consistent with our prior studies, ∼20% of brain AGO peaks were ‘orphans' lacking 6mer seed matches for the 35 most abundant miRNA families^22^. Chimera data linked miRNAs to 6,136 (∼28%) orphan peaks, disambiguating thousands of biologically robust non-canonical miRNA-binding sites.

Chimera-defined interactions and non-chimeric AGO CLIP reads were similarly distributed in the transcriptome (Fig. 1h). In addition to 3′-untranslated region (UTR) and coding DNA sequence (CDS) sites, chimeras identified many intronic sites with miRNA-dependent AGO binding (Supplementary Fig. 3a)^30^,^31^,^32^. Intronic interactions were not previously reported for CLASH in 293T cells, because reads were only aligned against mature transcripts^19^. Our alignment of raw CLASH data against a genomic reference recovered many intronic (∼15%) and other non-3′-UTR sites (>60%), independently confirming such binding. To examine whether annotated intronic interactions in the brain fall in mis-annotated exons, we examined polyA+ RNA sequencing from age-matched mouse cortex^33^. As polyA selection strongly enriches mature transcripts, introns show much lower coverage than coding or 3′-UTR exons. Accordingly, chimera-identified intronic sites showed low RNA sequencing coverage relative to exonic sites (Supplementary Fig. 3b). For comparison, binding sites for NOVA and RBFOX in the brain, which also bind intronic and exonic sequences, showed similar patterns^34^,^35^.

CLEAR-CLIP retrieved known miRNA regulatory sites (Fig. 1i and Supplementary Fig. 3c) and functions for well-characterized neuronal miRNAs, such as miR-124 and miR-9, in neuron development, synapse formation and axon guidance (Supplementary Data 3)^22^,^36^,^37^. Gene Ontology analysis indicated neuronal regulatory functions for less-characterized brain miRNAs, including miR-26 (for example, axon development and locomotion), miR-138 (neurotransmitter transport and secretion, and calcium transport) and miR-9* (cell migration and motility; Supplementary Data 3). In addition, Kyoto Encyclopedia of Genes and Genomes (KEGG) database analysis recovered known associations of miR-124, miR-9 and miR-26 with glioma, including known and many novel targets (Supplementary Fig. 4).

### CLEAR-CLIP-identified sites are functional

**Figure 2:**
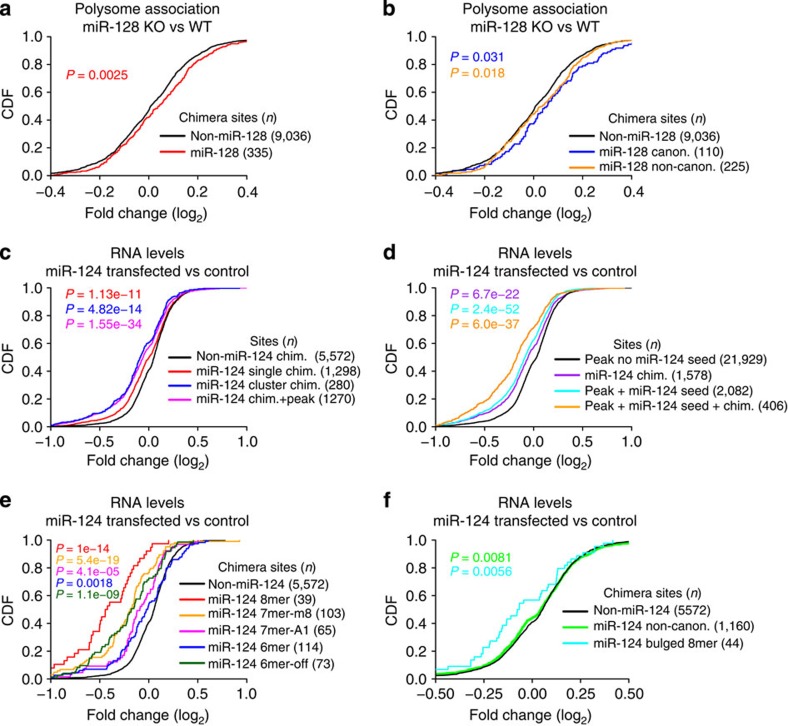
miRNA–target chimeras identify functional interactions. (**a**) Polyribosome association in miR-128 KO versus WT mouse brain^2^ plotted as a CDF for 3′-UTR sites identified with miR-128 chimeras (red) and non-miR-128 3′-UTR chimeras (black). (**b**) CDF as in **a** shown for canonical (blue) and non-canonical (orange) miR-128 sites. (**c**) Fold change in mRNA levels in CAD cells transfected with miR-124 mimic versus control^38^ are plotted as a CDF for 3′-UTR sites identified with miR-124 chimeras in the brain. miR-124 sites identified once (red), multiple times (blue) or overlapping AGO CLIP peaks (magenta) are shown compared with non-miR-124 sites (black). (**d**) CDF of 3′-UTR miR-124 sites as in **c**, showing miR-124 sites identified with chimeras (violet), peaks overlapping miR-124 seed matches (cyan) or peaks overlapping both seeds and miR-124 chimera(s) (orange). (**e**) CDF plots for transcripts with only chimera-defined canonical 3′-UTR miR-124 sites, broken down by site type. (**f**) CDF as in **e** for all 3′-UTR non-canonical sites (green) and bulged 8mer sites (cyan). In all panels, *P*-values from Kolmogorov–Smirnov testing comparing coloured subsets with control (black) sites are shown, along with the number of sites (*n*) in each set.

Chimera-identified sites from the brain are functional in global analyses of miRNA perturbation. For brain polyribosome-associated mRNAs from miR-128 knockout (KO) and wild-type (WT) mice, the presence of miR-128 chimeras in transcript 3′-UTRs correlated with enhanced polysome association in miR-128 KO brain (Fig. 2a)^2^. Sites with canonical seed matches and non-canonical sites predicted significant de-repression (Fig. 2b).

More detailed analysis was possible for miR-124 due to the large number of identified sites. In CAD neuroblastoma cells transfected with miR-124 mimic, the presence of miR-124 chimeras in 3′-UTRs in mouse brain correlated with repressed transcript levels compared with control cells (Fig. 2c)^38^. Chimera sites identified once (cluster size, *N*=1) predicted significant regulation and sites identified multiple times (*N*>1) or overlapping AGO CLIP peaks conferred stronger repression.

Consistent with our prior studies, AGO peaks encompassing miR-124 seed matches predicted significant transcript repression in miR-124-transfected cells (Fig. 2d)^22^. Critically, when such peaks overlapped miR-124 chimeras, repression was significantly greater. Thus, chimera information improved identification of functional miRNA sites *in vivo*. To examine different types of miR-124 sites, we defined mutually exclusive sets of transcripts possessing only chimera-defined canonical miR-124 sites or only non-canonical sites. Canonical sites correlated with significant transcript repression (Fig. 2e). Non-canonical sites predicted only a small shift in RNA levels (Fig. 2f) due largely to bulged 8mer miR-124 sites, the only non-canonical group predicting significant transcript repression in this data set. These analyses show that AGO HITS-CLIP maps supplemented with chimera data improved identification of functional miRNA target sites, including specific non-canonical sites.

### Diverse miRNA–mRNA pairing patterns

**Figure 3:**
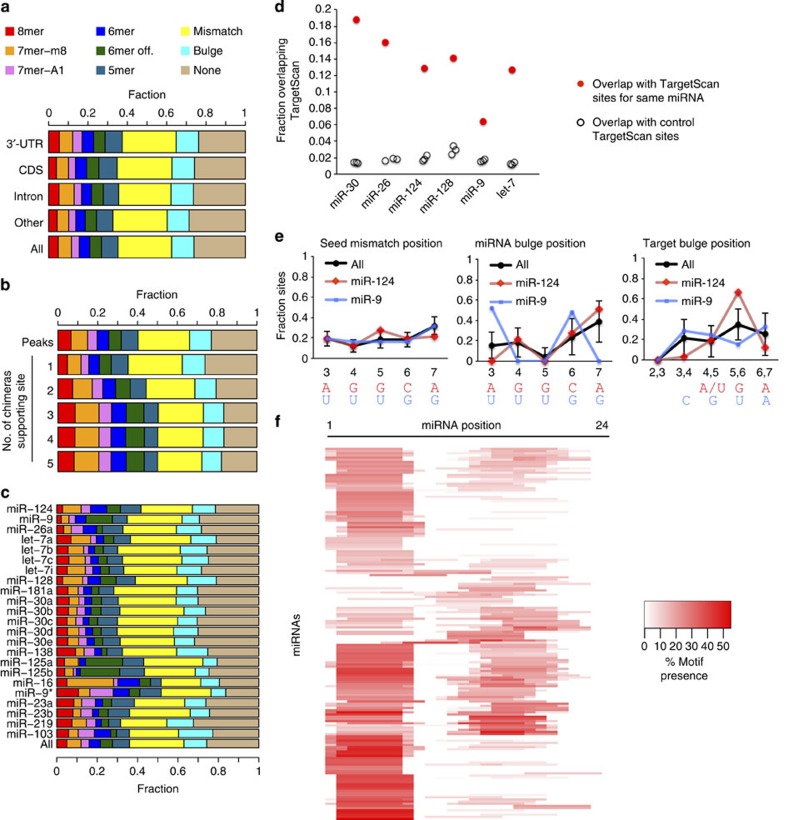
Motif analysis reveals miRNA binding dependent on seed and auxiliary pairing. The proportion of chimera-defined target regions with the indicated seed variants is plotted, broken down (**a**) by transcript region, (**b**) by the number of times interactions were identified with chimeras (*N*) or whether chimeras overlapped AGO CLIP peaks and (**c**) for the most abundant miRNA families in mouse brain, ranked from the top by decreasing abundance. (**d**) Overlap of 3′-UTR chimera-identified sites in the brain with TargetScan predicted sites for the same miRNA (red) or three equally sized random control sets of TargetScan sites (black). Control sets were restricted to the top 20 brain miRNAs. Only target sites in mRNAs with detectable expression in the cortex were considered. (**e**) The distributions of mismatched and bulged nucleotides for chimera-identified sites with imperfect seed motifs are plotted for the top 25 mouse brain miRNAs (black), miR-124 (red) and miR-9 (blue). Error bars show the s.d. at each position for the top 25 miRNAs in the brain. miRNA seed sequences for miR-124 and miR-9 are shown below mismatch and miRNA bulge plots. Below the target bulge plot, the most frequently bulged target nucleotide at the indicated position is shown when strong preferences (>50% of sites) were apparent. Sites from all transcript regions were included in this analysis. (**f**) *De novo* analysis of cognate miRNA-complementary-enriched 7mer motifs in all chimera target regions plotted as a heat map across the miRNA. Each line represents one miRNA and colour intensity scales with abundance in target sequences. miRNAs are ordered by hierarchical clustering.

In addition to canonical sites, motif searches allowing expanded seed match variants revealed a high proportion of single mismatch and bulged sites (>30% together), and many (∼20%) lacking appreciable seed homology (Fig. 3a). These patterns were similar across different transcript regions, showing that CDS and intronic AGO targeting follows similar rules to 3′-UTR binding. For chimera clusters of increasing sizes (*N*) and chimeras overlapping AGO peaks, canonical sites were slightly enriched (Fig. 3b). Similar canonical motifs were used by all miRNAs but relative frequencies varied (Fig. 3c).

We determined overlap of chimera-defined sites with TargetScan predictions, a purely bioinformatic approach, for six abundant brain miRNA families^7^. Chimera-identified sites in 3′-UTRs for a given miRNA were much more likely to overlap TargetScan-predicted sites for that miRNA than random control sites (Fig. 3d). Nonetheless, TargetScan supported only a minority of chimera-defined sites and concordance varied for different miRNAs. A major source of discrepancy was the preponderance of 6mer and imperfect seed match variants in chimera-identified binding sites, functional categories not present in TargetScan. Detailed analysis of imperfect seed sites confirmed established patterns, such as the miR-124 target G bulge between miRNA positions 5 and 6 (Fig. 3e)^13^. Other motifs revealed strong miRNA-specific preferences for the location of bulged miRNA or target nucleotides (Fig. 3e and Supplementary Fig. 5a,b). Notably, 22 of the top 25 brain miRNAs disallowed bulging at one or more sites, most often position 5 (16/25). These preferences identify specific single-nucleotide target deletions that, presumably by forcing unfavourable miRNA bulges, should effectively abolish AGO binding and regulation. Compared with bulged motifs, seed mismatches were more evenly distributed and showed less miRNA-specific variation (Fig. 3e and Supplementary Fig. 5c). An exception was G–U wobble interactions, which showed strong preferences such as miR-30 position 3 (Supplementary Fig. 3d).

Unbiased *de novo* motif analysis of chimera target regions identified strong enrichment of seed-complementary motifs (Fig. 3f)^39^. miRNAs without significant seed binding were mostly low-abundance, often passenger-strand isoforms, which could be affected by sampling error. In addition, many miRNA targets had strong enrichments for motifs complementary to miRNA 3′-end sequences. Several auxiliary motifs included the classic supplementary pairing region from nucleotides 13–16, but many different regions of auxiliary binding were evident^17^.

### Expanded miRNA–target pairing rules in the brain

**Figure 4:**
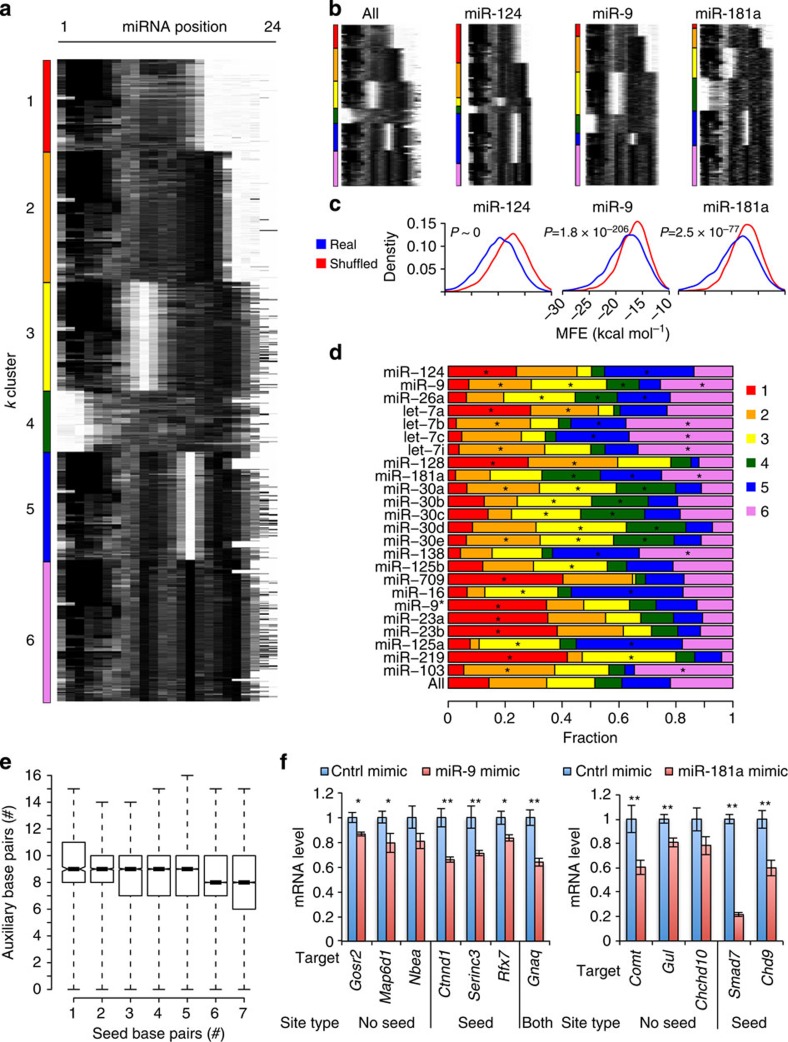
Duplex structure prediction reveals diverse targeting patterns for brain miRNAs. (**a**) RNAhybrid miRNA–target duplex structure predictions represented as heat maps^40^. Black pixels indicate base pairing and white pixels indicate gaps. Structures were partitioned by *k*-means clustering into six groups (see Methods). Interactions from all transcript regions were included in this analysis. (**b**) Structure maps for individual miRNAs compared with all. (**c**) Density plots of duplex minimum free energies (MFEs) are shown for the indicated miRNA–target interactions (blue) or shuffled interactions (red), where each chimeric target region was randomly re-assigned to an miRNA from a different chimeric interaction. MFEs were calculated with RNAhybrid. Axis labels are printed once, but apply to all plots. *P*-values from two-tailed *t*-tests are shown. (**d**) Distributions of the six identified *k*-clusters for the top brain miRNAs, ranked by decreasing abundance from the top to the bottom. Most brain miRNAs (∼90%) and all shown here have significant preferences versus the whole population (*positive enrichment, *P*<10^−3^, Fisher's exact test; full set is in Supplementary Table 3). (**e**) Box plot comparing number of predicted seed region base pairs with predicted auxiliary base pairs for all brain miRNA–target chimeras. (**f**) Experimental validation of chimera-identified seed-dependent and seedless (*k*=4, with no canonical seeds in 3′-UTR) miR-9 and miR-181a targets was performed by transfecting miRNA mimics into N2A cells and measuring endogenous targets by qRT–PCR. The average fold change in miRNA mimic versus control mimic-transfected cells is shown from four independent transfections, ±s.e.m. *P<0.05 and **P<0.01, one-tailed *t*-test. *Smad7*, a previously confirmed miR-181a target, served as a positive control^68^.

Motif analysis revealed extensive seed-based and auxiliary miRNA targeting *in vivo*. For resolution of individual events, we performed duplex structure predictions for target regions and their cognate miRNAs using RNAhybrid (Supplementary Data 1)^40^. *k*-means clustering of structures revealed six major modes of miRNA–target binding, with five dominated by seed-site pairing combined with various auxiliary binding patterns (Fig. 4a,b). Four clusters (*k*=1–4) closely mirrored similar analyses of 293T CLASH sites, including a seed-independent class (*k*=4)^19^. A fifth group identified by CLASH, encompassing ∼20% of interactions and lacking significant miRNA–target pairing, was not identified here. We also observed novel classes with seed pairing coupled with bipartite or tripartite auxiliary pairing patterns. These clusters, including the distinctive patterns of auxiliary binding, were not observed when target regions and miRNAs were shuffled by randomly re-assigning each chimeric target region the miRNA from a different chimera. Shuffled interactions showed significantly lower duplex hybridization energies than true ones, consistent with the discovery of real binding events (Fig. 4c).

Remarkably, of 212 miRNAs with >50 identified target sites in the brain, 196 (∼90%) showed significant enrichment or depletion in one or more *k*-means binding class (Fig. 4d and Supplementary Table 3). For example, miR-124 was strongly enriched in groups 1 (*P*=1.6 × 10^−245^, Fisher's exact test) and 5 (*P*<1.6 × 10^−245^), and marginally in group 2 (*P*=2.1 × 10^−3^). In contrast, miR-124 was strongly depleted in groups 3 (*P*<1.6 × 10^−245^), 4 (*P*=3.7 × 10^−140^) and 6 (*P*=1.1 × 10^−174^). This pattern confirmed strong seed dependence for miR-124 binding and revealed distinct patterns of favoured auxiliary binding (Fig. 4b,d). Motif analysis also supported auxiliary pairing, showing an enriched 7mer motif complementary to miR-124 positions 14 to 20 (Fig. 3f). Structural inference revealed distinct binding patterns contributing to this motif consensus.

Some miRNAs tolerated striking diversity in pairing interactions. miR-9 was enriched in group 3 (*P*=3.3 × 10^−130^, Fisher's exact test), characterized by strong seed dependence and frequent auxiliary pairing from positions 14 to 22, and group 6 (*P*=3.9 × 10^−17^), characterized by a tripartite auxiliary pattern (Fig. 4b). miR-9 was also enriched for seedless binding (*k*=4, *P*=2.2 × 10^−9^). Similarly, miR-181 family members were enriched in both seed-dependent and -independent classes. Globally, interactions with more predicted seed pairing exhibited fewer predicted auxiliary base pairs and vice versa (Fig. 4e). Canonical sites with less seed pairing (6mer and 7mer-A1) had slightly more predicted auxiliary pairing than stronger seed sites (8mer and 7mer-m8), consistent with supplementary 3′-pairing (Supplementary Fig. 6a)^17^. A stronger effect was evident for bulged or mismatched 8mer and 7mer motifs, which had more auxiliary pairing than their perfect match counterparts, indicating complementary pairing to offset imperfect seed matches (Supplementary Fig. 6b–d)^18^.

Specific classes of CLEAR-CLIP-defined sites are preferentially conserved in mammals, consistent with functional significance^7^,^41^,^42^. In both CDS and 3′-UTRs, groups 1, 2 and 3 were modestly more conserved than groups 4, 5 and 6, with seedless interactions (*k*=4) showing lowest overall conservation (Supplementary Fig. 7a,b). The 3′-UTR sites with canonical seed matches and certain bulged or mismatched motifs were more conserved than sites lacking seed homology (Supplementary Fig. 7c). CDS sites showed a similar pattern, except for mismatched sites (Supplementary Fig. 7d). To compare conservation of seed and auxiliary pairing regions, we calculated conservation scores in the seed and auxiliary portions of 3′-UTR target sites. For 8mer and 7mer-m8 sites, target seed regions were modestly more conserved than the auxiliary region (*P*<0.05, one-tailed *t*-test). For other sites, seed and auxiliary regions were similarly conserved (Supplementary Fig. 7e), implying evolutionary pressure to maintain the whole miRNA binding site.

We confirmed chimera-identified regulation by transfecting miRNA mimics into mouse neuroblastoma (N2A) cells and measuring endogenous target mRNA levels by quantitative reverse transcriptase–PCR (qRT–PCR). miRNA mimics repressed most miR-9 (6/7) and miR-181a (5/6) targets examined, including all with canonical seeds and several with seedless interactions and no canonical seed matches in their 3′-UTRs (Fig. 4f). These experiments support prior findings that seed-independent miRNA targeting is functional but weaker than seed-dependent regulation^14^,^19^.

### Endogenous miRNA–target chimeras in human hepatoma cells

**Figure 5:**
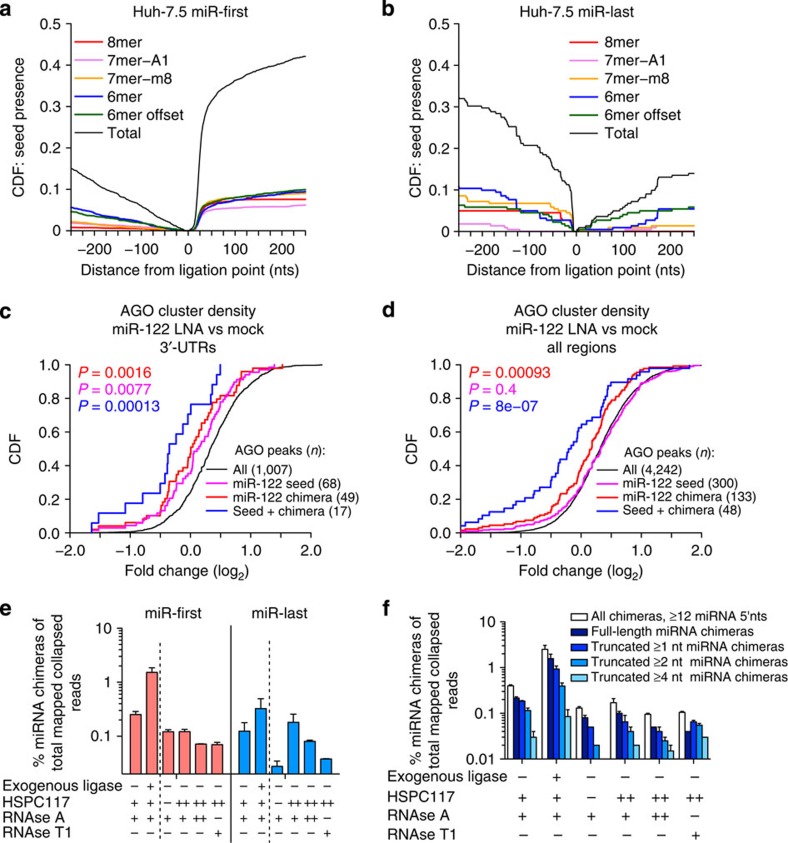
miRNA–target chimeras identify functional interactions in Huh-7.5 cells. CDF seed-enrichment plots as in Fig. 1d–g for miR-first (**a**) and miR-last (**b**) chimera target regions from Huh-7.5 HITS-CLIP. CDF plots of LNA-122 induced changes in AGO binding across 3′-UTRs (**c**) or all regions (**d**) for sites with miR-122 7-8mer seeds (magenta), miR-122 chimeras (red) or the combination of both (blue). *P*-values are shown for Kolmogorov–Smirnov tests comparing indicated subsets to control (black) sets. (**e**) miR-first and miR-last chimeras in CLEAR-CLIP on Huh-7.5 cells as a percentage of total unique reads, varying the presence of exogenous T4 RNA ligase and RNAse. For HSPC117 ligase, (+) represents endogenous levels, (−) represents siRNA knockdown and (++) represents overexpression as shown in Supplementary Fig. 8d. (**f**) Analysis of CLEAR-CLIP derived miR-first chimera truncation and the effects of HSPC117 manipulation. Percentage of chimeras harbouring full-length miRNAs was compared with chimeras with the indicated 3′-truncations, or with all putative chimeric reads with at least 12 nts miRNA sequence starting at the 5′-miRNA end. In **e**,**f**, the mean values of two biological replicates is shown for each sample, with error bars indicating s.d.

To independently assess miRNA–target pairing patterns, we searched for miRNA–target chimeras in standard HITS-CLIP libraries from human hepatoma (Huh-7.5) cells. miR-first chimeras were present at ∼0.5% of unique reads, suggesting that on-bead RNA ligase I treatment for 3′-linker addition in the standard protocol can form chimeras (Supplementary Table 4 and Supplementary Data 4). As in the brain, miR-first chimera target regions were strongly enriched for cognate miRNA seed matches, whereas miR-last were less so (Fig. 5a,b). In total, 34,986 miRNA–target interactions were identified in Huh-7.5 cells (Supplementary Fig. 8a)^43^, confirming that standard HITS-CLIP libraries contain miRNA–target chimeras, albeit at reduced frequency^26^.

To further test the functionality of chimera-identified sites, we examined data from Huh-7.5 cells treated with locked nucleic acid (LNA) against miR-122 or miravirsen, a clinical miR-122 inhibitor^44^. AGO binding to 3′-UTR regions with miR-122 7mer or 8mer seed matches was specifically reduced in miR-122 LNA versus control cells (Fig. 5c). This effect was stronger for sites overlapping miR-122 chimeras and even stronger when both predictors were combined. When regions outside 3′-UTRs were included, a significant effect was only observed when miR-122 chimeras were present (Fig. 5d). These results indicate that chimeras enhanced prediction of 3′-UTR and non-3′-UTR sites. For miravirsen treatment, miR-122 seed presence alone was predictive in all cases, but miR-122 chimeras enhanced these predictions (Supplementary Fig. 8b,c). This analysis provided further evidence that miRNA chimeras improve identification of miRNA regulatory sites.

### miRNA–target chimeras in the absence of exogenous ligase

Chimeras independent of exogenous ligase were present in small numbers in mouse brain and were reported in *C. elegans*^26^. These interactions showed significant seed enrichment, suggesting many are real (Fig. 1e). We used CLEAR-CLIP in Huh-7.5 cells to investigate mammalian transfer RNA ligase HSPC117 as a potential source of these chimeras and a means to enhance chimera ligation^45^. As in mouse brain, Huh-7.5 CLEAR-CLIP yielded chimeras at ∼2% of mapped reads. Ligase-treated samples showed a ∼10-fold enrichment for miR-first chimeras and a smaller enrichment for miR-last (Fig. 5e). CLEAR-CLIP without ligase addition was also done on Huh-7.5 cells with induced overexpression of HSPC117 or efficient depletion by RNA interference (Supplementary Fig. 8d). In both conditions, chimera frequencies were not significantly different from controls with endogenous HSPC117 levels (Fig. 5e). We also searched for chimeras containing truncated miRNAs, in case RNAse cleavage was a prerequisite for HSPC117-mediated ligation^26^, yielding the same result (Fig. 5f). Interestingly, truncated chimeras in Huh-7.5 cells comprised an additional ∼1% of mapped reads, far more than in the brain, with most truncated one nucleotide (Supplementary Fig. 8e). This analysis ruled out HSPC117 as a major endogenous source of chimeras.

### Expanded miRNA–target pairing rules in human cells

**Figure 6:**
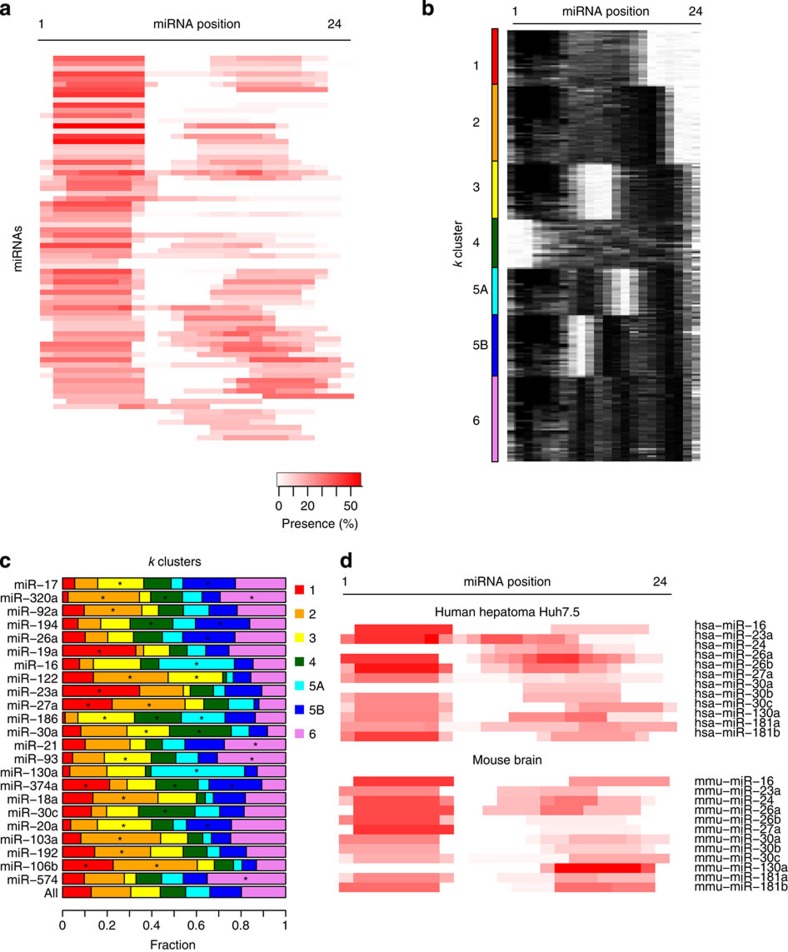
Expanded miRNA pairing rules for human miRNAs. (**a**) *De novo* analysis of cognate miRNA-complementary-enriched 7mer motifs in chimera target regions plotted as a heat map across the miRNA. Each line represents one miRNA, with colour intensity indicating abundance in target sequence. miRNAs are ordered by hierarchical clustering. Interactions from all Huh-7.5 HITS-CLIP and CLEAR-CLIP experiments from all transcript regions were included in these analyses. (**b**) RNAhybrid miRNA–target duplex structure predictions represented as heat maps as in Fig. 4a, partitioned by *k*-means clustering^40^. (**c**) Distributions of the seven identified *k*-clusters for top Huh-7.5 miRNAs ranked by abundance in chimeras from top to bottom. Most miRNAs (∼90%) and all shown here have distinct preferences versus the whole population. Interactions from all transcript regions were included in this analysis (*positive enrichment, *P*<10^−3^, Fisher's exact test; full results in Supplementary Table 5). (**d**) Comparative motif analysis heat map for the 12 miRNAs that were among the 50 most abundant in both mouse brain and Huh-7.5 cells.

Motif and structural analysis revealed global miRNA–target pairing patterns in Huh-7.5 cells. As in mouse brain, seed-complementary motifs were identified for most miRNAs, in addition to many 3′-auxiliary motifs (Fig. 6a). For structure clustering, informative binding classes in Huh-7.5 cells were most evident with seven *k*-groups, as opposed to six in mouse brain (Fig. 6b). Two Huh-7.5 groups (5A and 5B), similar to group 5 from mouse brain, showed bipartite auxiliary pairing but at distinct sites. The other clusters closely resembled corresponding groups in mouse brain. The appearance of more diversity in Huh-7.5 cells may reflect the diversity of their miRNA profiles, which included many miRNAs expressed at high to moderate levels (Supplementary Fig. 8f). Comparably, brain miRNA–target interactions involved fewer, very abundant miRNAs, consistent with a narrower range of structures (Supplementary Fig. 1e).

Of 83 human miRNAs detected in 50 or more chimeras, 75 (90%) were significantly enriched or depleted in specific binding classes (Fig. 6c and Supplementary Table 5). To assess the reproducibility of chimera-defined pairing patterns in different biologic settings, motif enrichments were compared for the 12 miRNAs among the 50 most abundant in both mouse brain and Huh-7.5 cells (Fig. 6d). Overall binding patterns were preserved across species and tissue types in 9 of 12 cases, supporting the robustness of our methods. The remaining three miRNAs showed similar enrichment of auxiliary motifs but divergent seed enrichments, which may reflect the different target populations in these settings.

### Auxiliary pairing regulates miRNA–target specificity *in vivo*

**Figure 7:**
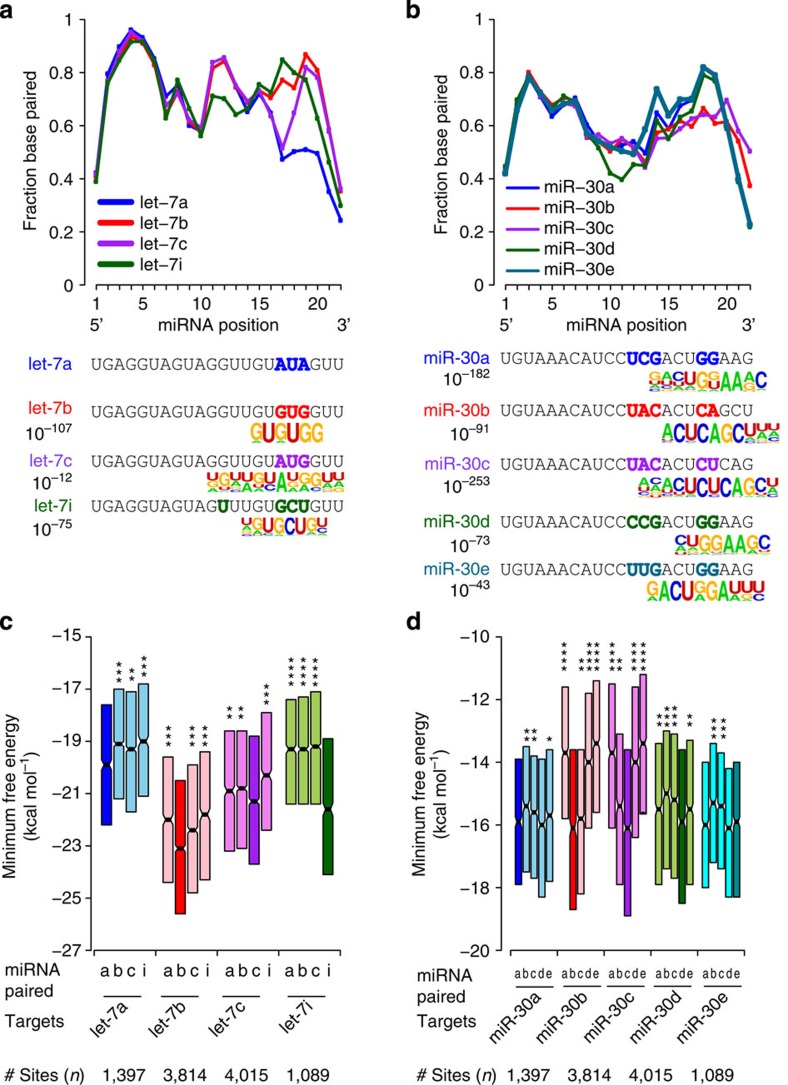
CLEAR-CLIP reveals target specificity among miRNA family members. Base pairing profiles from duplex structure maps for let-7 (**a**) and miR-30 (**b**) family members are shown. For each miRNA, the fraction of interactions with base pairing at each miRNA position is plotted. miRNA sequences are shown below with coloured bases indicating divergent nucleotides. *De novo* motif analysis of target regions for indicated miRNAs revealed family-member-specific motifs complementary to divergent parts of the miRNAs. For easier interpretation, the target motifs were reverse complemented to match the miRNA sequences. *P*-values for enrichment over background (AGO-binding regions in brain) from HOMER are indicated. No unique auxiliary motif was found for let-7a, the only such case. (**c**,**d**) Predicted minimum free energies (MFEs) from pairwise analysis of duplex structures for chimera-defined targets and the indicated let-7 (**c**) or miR-30 (**d**) family members is shown. Targets paired with their chimera-identified, cognate let-7 family member are shaded darker. Interactions from all transcript regions were included in these analyses. Box plots depict interquartile (25–75) values (*P<0.05, **P<0.001, ***P<10^−10^ and ****P<10^−50^, one-tailed *t*-test).

As a striking indication that auxiliary pairing regulates miRNA–target specificity, duplex structure analysis revealed distinct binding patterns for members of miRNA seed families (for example, let-7, miR-30, miR-181 and miR-125) (Fig. 4d). As CLEAR-CLIP does not yet provide comprehensive coverage of all miRNA-binding sites, it was not possible to compare the overlap of different miRNA paralogues by occupancy analysis. Instead, we used *de novo* motif analysis to search for distinguishing features of the target regions of individual paralogues. For most miRNA family members, motifs complementary to divergent 3′-sequences were highly enriched in cognate target regions but not their paralogues (Fig. 7a,b, below charts). Next, we reasoned that if inter-family preferences existed, family members should form more stable duplex structures with their own identified target regions than other paralogues. We calculated duplex energies for CLEAR-CLIP target regions of each abundant let-7 family member in the brain with each let-7 miRNA in a four-way pair-wise comparison (Fig. 7c). In all cases, let-7 family miRNAs formed more stable structures with their cognate target regions than other paralogues. This observation is striking in that some paralogues (for example, let-7b and let-7c) have higher GC content and thus intrinsic potential for more stable structures. Shuffling analysis of miR-30 family members revealed similar specificity, although certain preferences were more significant than others (Fig. 7d). Specifically, miR-30b and miR-30c showed more significant differences from miR-30a, miR-30d and miR-30e than from each other and vice versa. Analysis of miR-125 and miR-181 families revealed additional intra-family target preferences (Supplementary Fig. 9a–d). Thus, motif and structure information indicate distinct targeting preferences for miRNA paralogues controlled by differential miRNA 3′-end pairing.

**Figure 8:**
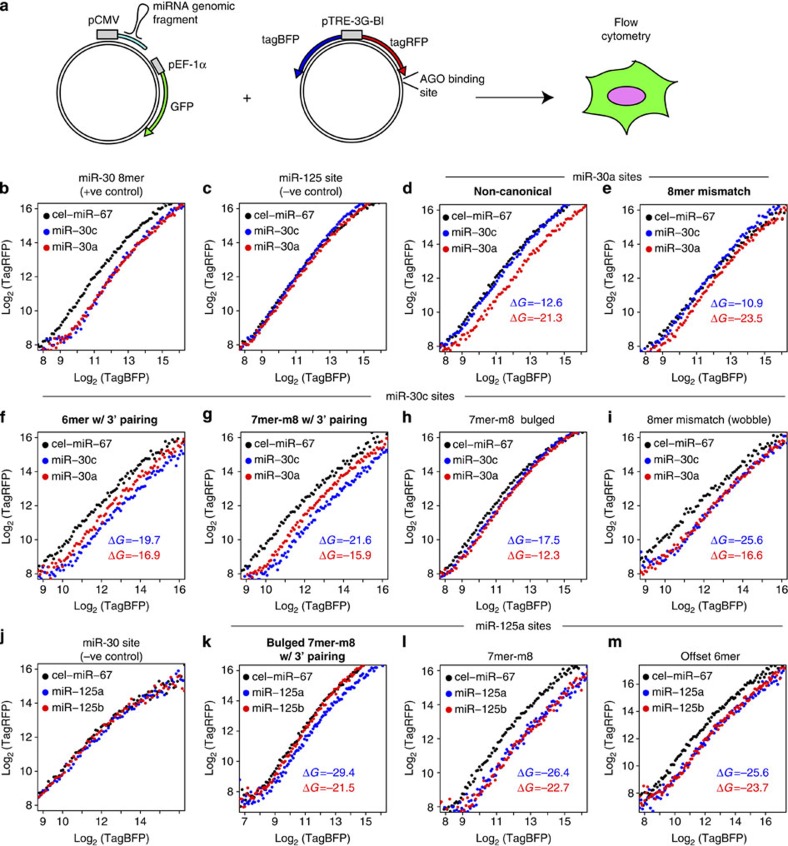
miRNA family member specificity confirmed by single cell measurements. (**a**) A system for single cell measurements of miRNA-mediated repression, adapted from ref. 46. tagRFP and tagBFP are expressed from the doxycycline-inducible bidirectional pTRE-3G-BI promoter. CLEAR-CLIP-defined AGO-binding sites were cloned into the 3′-UTR of the tagRFP cassette, with tagBFP used for internal normalization. Plasmids co-expressing miRNAs and GFP were co-transfected and measurements were taken 48 h later. (**b**–**m**) Log-transformed plots of tagRFP versus tagBFP fluorescence, with minimum free energies (MFEs) (ΔG) for predicted base pairing between duplex structures for indicated paralogues. A description of the site type is shown above each plot, with bold labelling denoting successful validation of paralogue specificity. Evaluation of miR-30a (red), miR-30c (blue) and negative control miRNA (black) overexpression on (**b**) a full miR-30 8mer site as a positive control for miR-30 paralogues; (**c**) a miR-125 site as a negative control for miR-30 paralogues; (**d**,**e**) sites with predicted miR-30a preference; and (**f**–**i**) sites with predicted miR-30c preference. Evaluation of miR-125a (blue), miR-125b (red) and negative control miRNA (black) overexpression on (**j**) a miR-30 site as a negative control for miR-125 paralogs and (**k**–**m**) sites with predicted miR-125a preference. Representative plots from at least two independent experiments for each construct are shown.

We validated functional specificity of miRNA family members using fluorescence reporters with paralogue-specific target sites in their 3′-UTRs (Fig. 8a)^46^. We examined miR-30a, miR-30c and miR-125a targets sites predicted to form more stable pairing with a specific paralogue and which were ligated to only that paralogue in at least two CLEAR-CLIP experiments. Reporters were co-transfected into N2A cells with plasmids expressing miRNA family members or a control *C. elegans* miRNA. miRNA expression was confirmed by northern blotting (Supplementary Fig. 10a) and silencing activity was confirmed using reporters with perfect complementary sites (Supplementary Fig. 10b,c). For CLEAR-CLIP-defined sites, repression was specific or more significant for the predicted paralogue in several cases (Fig. 8d–g,k). Effects included supplementary 3′-pairing enhancing canonical repression (Fig. 8f,g) and paralogue-specific regulation at non-canonical sites (Fig. 8d,e,k). For other sites, repression in the presence of canonical (Fig. 8l,m) or non-canonical (Fig. 8h,i) sites was similar for different family members. When predicted pairing for one paralogue was significantly more stable (> 6 kcal mol^−1^ Δ minimum free energy), paralogue-specific activity was usually observed. An exception was an 8mer mismatch miR-30c site with G–U wobble pairing at miRNA position 3, which showed similar repression by both miR-30a and miR-30c despite extensive predicted 3′-pairing with miR-30c (Fig. 8i). The strong repression by both paralogues was comparable to that of a perfect 8mer site (Fig. 8b), consistent with our finding that G–U pairing is well-tolerated at specific seed positions (Supplementary Fig. 5d). Conversely, more subtle differences in predicted pairing (2.8 kcal mol^−1^) enhanced miR-30c activity at a 6mer site with predicted supplementary 3′-pairing (Fig. 8f). This complexity underscores the need for empirical binding maps to supplement structure- and sequence-based predictions. More broadly, these results illustrate paralogue-specific miRNA activity and diverse functional classes of non-canonical sites.

## Discussion

CLEAR-CLIP gains its power from the formation of sequential covalent bonds that reflect *in vivo* interactions. The utility of miRNA–target chimeras was demonstrated in two prior studies using CLASH and *in vivo* photoactivatable ribonucleoside-enhanced CLIP^19^,^26^. In mixing experiments, CLEAR-CLIP showed low false target identification rates similar to these approaches without relying on specialized tagging strategies. CLEAR-CLIP thus provides a snapshot of true, physiologic miRNA–target interactions and is uniquely applicable to all mammalian model systems and human samples^47^. In contrast to CLASH, CLEAR-CLIP does not require fully denaturing AGO and involves a single purification step. Our experiments with denatured AGO and analyses of published CLASH data showed low yield of standard non-chimeric CLIP reads compared with standard AGO HITS-CLIP, hindering robust AGO-binding peak identification. With straightforward modifications of HITS-CLIP, CLEAR-CLIP simultaneously generates chimera information and high-quality, transcriptome-wide AGO HITS-CLIP maps. These dual data sets improved identification of functional miRNA target sites compared with HITS-CLIP or chimeras alone (Figs 2d and 5c,d), a key advantage, as miRNA–target ligation remains limiting. Optimized ligation conditions yielded at least tenfold enrichment in ligase-treated versus no-ligase samples, a substantial improvement over prior methods^26^, but insufficient for comprehensive coverage. A key future goal is further improvement of this efficiency to reduce false negatives and achieve the global coverage of HITS-CLIP maps.

CLEAR-CLIP yielded insights into pairing rules for over 200 mammalian miRNAs. Enriched target motifs revealed seed-dependence for most miRNAs, with widespread bulged or mismatched pairing, and extensive 3′-auxiliary interactions (Figs 3 and 6). miRNA–target duplex structure prediction clarified that most interactions employed seed and auxiliary pairing in combination (Figs 4 and 6). Most miRNAs were significantly enriched or depleted in one or more binding class, with many favouring two or more categories. This tolerance for distinct but constrained pairing structures was most apparent for abundant miRNAs with robust maps, suggesting that increased CLEAR-CLIP and CLASH efficiency and/or profiles in additional cell types will reveal similarly diverse pairing rules for other miRNAs. Similar pairing patterns applied to conventional 3′-UTR targeting, as well as CDS and intronic binding. The latter indicates extensive, miRNA-dependent nuclear targeting of AGO. Although previous studies established AGO nuclear localization and RNA binding^22^,^30^,^31^,^48^, its mechanistic dependence on miRNA guidance was previously unclear.

Motifs and structure inference showed extensive pairing of miRNA 3′-ends with targets. Such auxiliary interactions can stabilize or enhance miRNA–target pairing, in particular together with imperfect seed pairing^18^. Global analysis of bulged and mismatched seed interactions from CLEAR-CLIP shows this phenomenon is common (Supplementary Figs 5 and 6). The importance of 3′-auxiliary binding is still debated, with some reports demonstrating significant effects^18^,^49^ and others concluding limited ones^7^. Analyses of miRNA mimic transfections found that supplementary pairing of miRNA bases 12–17 marginally enhanced target repression in rare instances^17^,^50^. However, the sensitivity of such analyses may be limited by stringent requirements for continuous spans of auxiliary binding^7^. CLEAR-CLIP revealed diverse, often discontinuous auxiliary pairing that could hinder the detection of motif presence or conservation above background (Figs 4a and 6b). A second consideration is the heavy reliance of prior conclusions on acute overexpression of miRNAs, which may perturb endogenous AGO–miRNA–target stoichiometry or interrogate different target repertoires than are available *in vivo*. Recent evidence for co-evolution of miRNAs and targets, in particular in neurons, underscores the importance of examining physiologic interactions^51^. The use of transcript destabilization *in vitro* as a sole functional readout may also overlook other AGO functions, including translational control, targeting to non-3′-UTR regions and interactions with other RNA-binding proteins^42^.

As a striking indication that auxiliary interactions regulate miRNA target specificity, we observed specificity among paralogues in miRNA seed families (Fig. 7). Such specificity was previously illustrated for two let-7 family targets in *Drosophila* and has been speculated elsewhere^18^. Functional single-cell assays confirmed paralogue specificity for several sites from brain CLEAR-CLIP (Fig. 8). Other sites were similarly regulated by different paralogues, indicating miRNA family members are functionally redundant at certain sites and specific at others. Indeed, the strict conservation of miRNA families and their unique expression patterns *in vivo*, including across brain regions, supports specific functions^52^,^53^.

The predominance of canonical seed pairing in mediating mRNA target level repression is supported by CLEAR-CLIP-defined sites (Fig. 2). In addition, CLEAR-CLIP data demonstrated widespread, functional non-canonical miRNA targeting and substantial diversity in canonical and non-canonical interactions among different miRNAs. CLEAR-CLIP identified functional, non-canonical regulation globally for miR-128 and miR-124 (Fig. 2), and for individual miR-9, miR-181, miR-30 and miR-125 targets (Fig. 4f and Fig. 8b–m). Non-canonical sites included diverse seed mismatch and bulged variants, and seedless interactions in both mouse brain and Huh-7.5 cells. Interestingly, a number of major miRNAs enriched for seedless interactions (for example, miR-9, miR-181, miR-30 and miR-186) have AU-rich seed sites, indicating that weak seed-pairing stability may favour seedless non-canonical interactions^10^. Our results support growing evidence of widespread non-canonical miRNA regulation that is likely to have a large collective impact^13^,^14^,^15^,^17^,^19^,^20^,^21^. We expect CLEAR-CLIP and similar methods will facilitate discovery of these sites and refine *in vivo* miRNA regulatory maps in future studies.

## Methods

### Mice

All mouse experiments were approved by The Rockefeller University Institutional Animal Care and Use Committee regulations. P13-aged C57BL6/J mice were used for all experiments, except for BR21, BR22 and BR23 (*Drosophila* mixing), which used 6-week-old mice.

### CLEAR-CLIP

*Tissue cross-linking and lysis.* Neocortex was dissected and cross-linked as described and snap frozen^54^. Frozen pellets were re-suspended in threefold volume (w/w) lysis buffer (1 × PBS/1% Igepal/0.5% sodium deoxycholate/0.1% SDS) containing Complete protease inhibitors (Roche). Lysates were treated with 30 μl RQ1 DNAse (Promega) at 37 °C for 5 min with shaking.

*Pre-immunoprecipitation RNAse treatment.* For samples BR1, BR2, BR4, BR13, BR14, BR15, BR16, BR17, BR18, BR19, BR20, BR21, BR22 and BR23, RNAse A (USB Products) was added to lysates at 0.0001 U μl^−1^ and incubated at 37 °C for 5 min. RNAsin Plus (Promega) was added at 0.5 U μl^−1^ and lysates were cleared by ultracentrifugation (50 000*g*). For remaining samples, RNAse treatment was done after immunoprecipitation (see below).

*Immunoprecipitation and washing.* Cleared lysates were rocked with Dynal Protein A beads (Life Technologies) prepared with 2A8 anti-AGO^27^ for 90 min at 4 °C, then washed:

- Three times lysis buffer containing 5 × Denhardt's solution
- Twice high-detergent buffer (1 × PBS/1% Igepal/1% sodium deoxycholate/0.2% SDS).
- Three times low-salt buffer (15 mM Tris pH 7.5, 5 mM EDTA)
- Twice high-salt buffer (1 × PBS/1% Igepal/0.5% sodium deoxycholate/0.1% SDS, 1 M NaCl (final, including PBS)).
- Twice PNK wash buffer (50 mM Tris pH 7.5, 10 mM MgCl_2_, 0.5% Igepal)

*On-bead RNAse treatment.* For samples BR3, BR5, BR6, BR7, BR8, BR9, BR10, BR11 and BR12, beads were re-suspended in 0.5 ml lysis buffer containing 2 mg ml^−1^ BSA and RNAse A at 0.00002 U μl^−1^. Samples were treated at 37 °C for 5 min with shaking, transferred to ice and supplemented with 0.5 U μl^−1^ RNAsin Plus. Beads were rocked for 20 min at 4 °C, to recover any dissociated antigen, then washed:

- Twice high-detergent buffer
- Three times low-salt buffer
- Once high-salt buffer
- Twice PNK buffer

*5′-End phosphorylation and chimera ligation.* Beads were treated with PNK (3′-phosphatase minus) (NEB) and 1 mM ATP to phosphorylate cleaved mRNA 5′-ends. Beads were washed three times in PNK buffer, then chimera ligation was performed overnight at 16 °C with 0.625 U μl^−1^ T4 RNA Ligase I, 1 mM ATP and 0.1 mg ml^−1^ BSA in a 100 μl total volume. The following morning, fresh RNA Ligase I (25 U) and ATP (1 mM) were added to each sample and incubation was continued 4–6 h. For minus-ligase controls (BR4 and BR5), RNA ligase was omitted. Beads were washed:

- Twice lysis buffer
- Once PNK/EDTA/EGTA buffer (50 mM Tris pH 7.5, 10 EDTA,10 mM EGTA, 0.5% Igepal)
- Twice PNK buffer

*Alkaline phosphatase treatment and 3′-linker ligation.* Alkaline phosphate treatment was performed to remove 3′-phosphate groups^27^. Pre-adenylated 3′-linker (5′-rAppGTGTCAGTCACTTCCAGCGG-3′) was added using truncated RNA Ligase 2 (NEB), with 2.5 μl 20 μM linker and 4 U enzyme per 40 μl reaction (16 °C overnight).

*Radiolabelling of AGO–RNA complexes.* AGO–RNA complexes were radiolabelled directly with PNK treatment in the presence of [γ-^32^P]-ATP, followed by cold chase, exactly as described^27^.

*SDS–PAGE and amplification of RNA footprints.* SDS–PAGE, nitrocellulose transfer, extraction of AGO-bound RNA, 5′-linker ligation and RT–PCR steps were performed exactly as described^27^.

*Addition of high-throughput sequencing adapters*. Adapters for high-throughput sequencing were added to libraries with additional PCR cycles. PCR conditions were exactly as described, but indexed primers specified in Supplementary Table 7 allowed sample multiplexing. Libraries were sequenced on the Illumina Hiseq 2500 platform with 100-nucleotide single-end reads or on the Illumina Miseq with 75-nucleotide single-end reads.

*CLEAR-CLIP with AGO denaturation*. AGO–RNA complexes were purified as described up through PNK treatment, then eluted from beads with denaturation buffer (50 mM Tris pH 7.5, 0.1% Igepal, 6 M guanidine HCl, 300 mM NaCl). Samples were diluted fivefold in 1 × PBS/0.1% Igepal and run over a buffer exchange column (Pierce) equilibrated with lysis buffer. AGO–RNA complexes were re-captured on fresh beads conjugated to 2A8 antibody, which was confirmed by western blotting. Subsequent steps were performed as described above.

*CLEAR-CLIP mixing experiments*. Total *E. coli* RNA was isolated with the RNAsnap method^56^. Either equal amounts or a sixfold excess of *E. coli* RNA (by mass) was equilibrated in lysis buffer and added to brain lysates. CLEAR-CLIP was then performed exactly was described, starting with DNAse treatment. For analyses in Supplementary Fig. 2, RNA was extracted after DNAse treatment (with or without RNAse) with Trizol LS and analysed by Bioanalyzer (Agilent) and qRT–PCR. For *Drosophila* mixing experiments, lysates from non-cross-linked S2 cells and cross-linked mouse brain containing equal mass amounts RNA were combined immediately post lysis and CLEAR-CLIP was performed starting at DNAse treatment.

*CLEAR-CLIP in Huh7.5 cells*. Huh7.5 CLEAR-CLIP was done as above with the following modifications. Cells (2 × 10^7^) growing in 150 mm plates were irradiated once for 400 mJ cm^−2^ and once for 200 mJ cm^−2^ using a Spectrolinker XL-1500 (Spectronics Corporation). Cells were trypsinized, pelleted and stored at −80 °C. Lysis was done in 1 ml lysis buffer. RNAse A (0.0004–0.00004 U μl^−1^; see Supplementary Table 4) or 0.1 U μl^−1^ RNAse T1 (Ambion) was used for RNAse treatment.

*AGO HITS-CLIP in Huh7.5 cells*. Standard AGO CLIP was done as per the previously published protocol^27^, except for multiplexing modifications described above.

### Plasmids

pRetroX-TRE3G-HSPC117 plasmid was constructed by inserting the HSPC117 (c22orf28) sequence from pLX304-c22orf28-H9 (ref. 45) into the doxycycline-inducible retroviral vector pRetroX-TRE3G (Clontech).

The dual-colour reporter vector was described elsewhere^57^. Inserts corresponding to CLEAR-CLIP-defined binding sites were synthesized as gBlocks (IDT) (Supplementary Table 6) and cloned into the 3′-UTR of tagRFP by Gibson Assembly (NEB) using EcoRV-linearized vector and inserts at a 1:5 molar ratio. Transformed clones were grown as maxi-preps at 30 °C and confirmed by restriction digests and sequencing.

Mouse miR-125a construct was purchased from SBI (MMIR-125a-PA-1). Genomic fragments for miR-125b, miR-30a and miR-30c spanning ∼200 nucleotides upstream and downstream of primary hairpins were synthesized as gBlocks (IDT) and inserted into the SBI vector between EcoRI and BamHI. Constructs expressing miR-30a from the miR-30c locus and miR-125b from the miR-125a locus were also made, in an effort to control for processing efficiency. However, miR-30a was only expressed from its endogenous locus (Supplementary Fig. 10). Therefore, endogenous fragments were used in all reporter experiments. The cel-miR-67 hairpin was cloned into the miR-30c genomic locus. Efficient expression of cel-miR-67 was confirmed by qRT–PCR using the miScript system (not shown).

### Cell culture and transfections

N2A mouse neuroblastoma (ATCC) and Huh7.5 human hepatoma cells^58^ were maintained in standard conditions.

N2A miRNA mimic ‘reverse' transfections were done with Dharmafect1 reagent and miRIDIAN mouse miRNA mimics or negative control mimic #1 (Dharmacon). Complexes were pre-formed in 24-well dishes, according to manufacturer's instructions, and 120 000 cells per well were added giving a final mimic concentration of 25 nM.

To generate N2A cells stably expressing the Tet-3G activator construct (Clontech), N2A cells were transfected with Xtremegene 9 (6:1 reagent:plasmid ratio, 375 ng plasmid per 24-well) and split at varying dilutions into G418 media 48 h later. Functional clones were identified by transfecting pTRE-BI-RFP construct and screening for doxycycline-inducible red fluorescent protein (RFP) expression.

For inducible expression of HSPC117, Huh7.5 cells expressing Tet-3G activator (kind gift from C. Takacs) were transduced with pRetroX-TRE3G-HSPC117. HSPC117 expression was induced by 3 μg ml^−1^ doxycycline.

For Huh7.5 cell miRNA inhibitor experiments, cells were seeded the day before and transfected with LNA-122 or miravirsen/SPC3649 (5′-CcAttGTcaCaCtCC-3′; LNA in upper case and DNA in lower case, Exiqon) at 30 nM using RNAi/Max (Life Technologies). No significant cytotoxicity was observed from the applied concentrations of LNA and miravirsen/SPC3649, as determined using CellTiter-Glo (Promega).

### qRT–PCR analysis

For miRNA mimic experiments, RNA was extracted from N2A cells 24 h post transfection with Trizol (Ambion). RNA was further purified with DNAse treatment on High Pure RNA Isolation columns (Roche). Total RNA (0.5 μg) was reverse transcribed with the iScript kit (Biorad). qPCR was done with SYBR Green Mix (Life Technologies) on the iQ Cycler (Biorad). Gene-specific primers (Supplementary Table 7) were designed with Primer3 and tested to confirm efficient amplification of single products^59^. The following programme was carried to 40 cycles: 30 s 95 °C (denaturation); 30 s 58 °C (annealing); and 20 s 72 °C (extension). Results were analysed by ΔΔCt, using *RPL10A* mRNA, an abundant transcript with negligible AGO binding in its 3′-UTR in brain, for normalization.

For *E. coli*/mouse mixing experiments in Supplementary Fig. 2, RNA was extracted with Trizol LS (Ambion). Equal volumes re-suspended RNA were reverse transcribed with the iScript kit and analysed by qPCR as above.

### Western blotting

For western blottings, 10 μg protein from cleared Huh-7.5 lysates were run per lane of a 4–12% NuPage gel (Life Technologies) and blotted onto a polyvinylidene difluoride membrane. HSPC117 was detected using Anti-C22orf28 antibody (Abcam, ab98231, 1 μg ml^-1^) and Goat-anti-Rabbit-HRP (Pierce 31462, 1:50,000).

### Flow cytometry

N2A-Tet3G cells were co-transfected with miRNA (250 ng) and reporter (125 ng) plasmids in media with 1 μg ml^−1^ doxycycline (Sigma). At 24 h media was refreshed and at 48 h cells were trypsinized, harvested and fixed with Cytofix/Cytoperm buffer (BD Biosciences). Cells were analysed on the MACSQuant cytometer (Miltenyi Biotec). Data were processed as described^46^,^57^. Briefly, single cells were gated in FlowJo software and fluorescence values were exported for analysis with custom R scripts. Cells were binned on the basis of tagBFP fluorescence and mean tagRFP fluorescence was calculated for each bin. Binned tagRFP means were plotted against binned tagBFP means.

### Northern blotting

RNA was extracted from transfected N2A cells or brain with Trizol. Thirty micrograms of RNA per sample were run on 15% urea PAGE gels and then transferred to nylon membranes (Perkin Elmer). Hybridization of ^32^P-labelled DNA oligonucleotide probes (Supplementary Table 7) was done at 37 °C in Ultrahyb-Oligo buffer (Ambion) overnight. Membranes were washed four times with 2 × SSC/0.1% SDS and exposed to film.

### Bioinformatic analysis

Initial bioinformatic processing was performed exactly as described^27^. An additional de-multiplexing step was added after 3′-adapter removal using a simple search for sample-specific indices (Supplementary Table 1). Peak calling for brain AGO HITS-CLIP was done as described, using pooled reads from ten biological samples in the present study and five from a prior one^22^.

*Identification of miRNA–mRNA chimeras*. Reads containing miRNA sequences were identified by ‘reverse' mapping mature miRNA sequences against sample libraries using Bowtie^60^. Changes to default parameters were as follows: maximum mismatches allowed in the seed (−*n*=1), seed length (−*l*=8), maximum total of quality values at mismatched read positions (−*e*=35) and maximum reported alignments (−*k*=−1). Reads mapped to more than one miRNA, usually members of the same miRNA family, were collapsed to a single, randomly chosen hit for initial analyses. Chimeric sequences upstream (5′) and/or downstream (3′) of miRNAs were extracted, filtered for a minimum length of 18 nt and mapped against the appropriate reference genome (mouse mm9, human hg18, *Drosophila* dm3 or *E. coli* (Genbank CP000948.1)) with Bowtie. Only single, uniquely mapped hits were allowed and PCR duplicates were consolidated as described^27^. Fragments mapping to miRNA genes were removed.

miR-first chimeras in the brain were present in ∼14-fold excess of miR-last (Supplementary Table 1). This result differs from reported CLASH results, where miR-first and miR-last species were present at comparable levels^19^. This difference may reflect an idiosyncrasy of AGO1, the only AGO paralogue analysed by CLASH, or denaturation of AGO in the CLASH protocol, which may expose the buried miRNA 5′-end. In CLEAR-CLIP, miR-last chimeras frequently involved dubiously annotated miRNAs, did not reflect endogenous miRNA abundance and were not formed by exogenous ligase. They were therefore excluded from subsequent analyses. Unique miR-first chimeric reads linked to same miRNA and with overlapping genomic coordinates were clustered together, using the GenomicRanges package in R^61^.

*Analysis of chimera targets in miRNA perturbation experiments*. Normalized microarray values for polyribosome profiles in miR-128 KO and WT mouse brains were obtained from GEO^2^. Genes with contradictory probe information (different signs) were filtered and probe log_2_ fold-change (log_2_FC) values for remaining genes were averaged. For cumulative distribution function (CDF) analysis (Fig. 2a,b), log_2_FC ratios (KO/WT) in transcript polysome association were plotted for miR-128 3′-UTR chimera sites. Non-miR-128 3′-UTR chimeras were plotted as controls.

Normalized microarray values for CAD neuroblastoma cells transfected with miR-124 or control mimics were obtained from GEO and processed as for miR-128 profiles^38^. In Fig. 2c, transcripts were divided into mutually exclusive sets based on the number of times (*N*=1 or *N*>1, where *N* is the number of times an interaction was identified by CLEAR-CLIP) the most frequently identified chimera site in their 3′-UTRs occurred. Log_2_FC ratios (miR-124/control) were plotted as CDFs. miR-124 sites overlapping AGO-binding peaks, regardless of cluster size (*N*), were also plotted. The control set (non-miR-124, black) for all analyses were sites from transcripts lacking miR-124 chimeras. In Fig. 2d, CDFs were plotted for chimera-identified miR-124 sites, peak-identified sites overlapping miR-124 seed matches and the intersection of those sets. In Fig. 2e,f, transcripts were divided into mutually exclusive sets based on the presence of only canonical miR-124 chimera sites (e) or only non-canonical sites (f) in 3′-UTRs.

For LNA-122- or miravirsen-treated Huh7.5 cells, standard AGO CLIP data from four biological replicates each of mock, LNA-122 and miravirsen were analysed, with alignment and peak calling as described above. Clusters were normalized to the read depth of their respective libraries after adding a pseudo-count of 1. Canonical miRNA seed searches were carried out within robust AGO clusters (±32 nts). AGO clusters overlapping miRNA chimeras were identified with the genomeIntervals R package^30^. For the CDF plots shown, a minimum BC of 4 and a cluster density of 40 was required.

*Sequence extraction and analysis*. Sequence extraction and seed motif searches, including for mismatch and indel variants, were done with the GenomicRanges and BioStrings packages in the R Bioconductor suite^61^,^30^. Only single-nucleotide mismatches or indels were allowed. Clustered target regions up to 75 nt downstream of the ligation site, which sometimes extended beyond the sequenced reads, were searched. The selection of this interval was based on our observation that the vast majority of 8mer and 7mer-m8 seed matches fell within this region. These 75 nt regions were used subsequently for motif and structure analysis.

*TargetScan 6.2 overlap*. Genomic coordinates for mouse TargetScan 6.2 sites were filtered for genes expressed in P13 cortex^7^. Per cent overlap of 3′-UTR CLEAR-CLIP regions for the indicated miRNAs (collapsed by seed family, Fig. 3d) and TargetScan sites for that miRNA was calculated. For each miRNA, overlap was also calculated for three negative control sets of equal size, randomly selected from TargetScan sites for the top 20 abundant miRNAs (also only in cortex-expressed transcripts).

*Motif analysis*. For *de novo* motif analysis in chimera target sequences, chimeras were grouped for each miRNA present in at least 50 individual chimeras and 40 individual sites. Background sequences totaling five times the number of foreground (target) sequences were selected from other miRNA chimeras, excluding other miRNAs with the same seed site. *De novo* motif discovery was performed on three independent background sets using Homer^39^, expecting 7mer motifs and checking motifs for complementarity to the cognate miRNA, using commands similar to:

perl bin/findMotifs.pl foreground/hsa-miR-122-5p.txt fasta output/hsa-miR-122-5p/ -fasta background/hsa-miR-122-5p.txt -mcheck motifs/hsa-miR-122-5p.motif -norevopp -noknown -len 7 -bits

Reverse complement miRNA sequences were added to the Homer list of known motifs using commands similar to:

perl bin/seq2profile.pl CAAACACCATTGTCACACTCCA 0 hsa-miR-122-5p > motifs/hsa-miR-122-5p.motif

Information from Homer output files was extracted using regular expressions in R and a combined confidence parameter, *c*, was calculated as:

*c*=(−log_10_(*p*)−10)/10+(*s*−0.35) × 6.7,

where *p* is the *P*-value and *s* is the match score with the given miRNA from Homer. Motifs with *s*≥0.35, information content per bp≥1.75 and *c*≥1 were retained. In seven iterations of random comparisons of background sequences, *P*-values below 1*e*−10 were rarely observed and *c*-values meeting the threshold were never observed. Heat maps were created in the R gplots package.

*RNA duplex structure prediction*. Duplex structure predictions for miRNA and target region were made with RNAhybrid^40^. The first miRNA nucleotide was trimmed, as this position does not basepair to targets^62^. Target regions (75 nt) were examined. Clusters>100 nt in length (<0.5% of total) were omitted. Clusters >75 nt and ≤100 nt were trimmed symmetrically from both ends to a length of 75 nt.

We reasoned that canonical seed matches and variants were likely to be engaged in base pairing when present. Default RNAhybrid settings identified most seed matches in target regions (∼71% of total and ∼80% of 8mers). To improve concordance with motif presence, pairing was forced at appropriate seed positions when 8mer, 7mer, 6mer or 5mer matches were present, improving concordance to ∼95%. For targets with mismatch (8mer, 7mer and 6mer) or bulged (8mer and 7mer) motifs, two duplexes were predicted with forced pairing at positions 3 and 4 (setting –f 3,4) or positions 5 and 6 (–f 5,6). Predicted structures were usually identical, but when different the lower energy structure was used. For targets lacking seed homology, seed pairing was not forced (–f option omitted).

For duplex heat maps, base-paired (Watson–Crick or G:U) miRNA sites were assigned a score of 1 and unpaired sites a score of 0. *k*-means clustering of the resulting matrix was done with Cluster 3.0 and visualized with Java TreeView^63^,^64^. Cluster numbers (*k*) 3–12 were tested, with *k*=6 providing the most meaningful set of distinct categories in the brain. Enrichments of miRNAs in different k groups were evaluated by Fisher's exact test, comparing the distribution of each miRNA against all interactions. Analyses for Huh7.5 data were done identically, but *k*=7 yielded more intuitive clustering of interactions.

*Conservation analysis*. Conservation scores (phlyoP) for duplex regions defined by RNAhybrid were downloaded from UCSC Genome Browser^65^,^66^. Plotted conservation scores for target regions were calculated by averaging base-wise phyloP scores across intervals.

*Analysis of miRNA family specificity*. To remove ambiguity in assigning chimeras among family members, Bowtie alignments were repeated with no mismatch allowance. For miRNA base-pairing profiles, the percentage of chimera-identified interactions with base pairing at each miRNA position was calculated from duplex map predictions. For pairwise comparisons of predicted structures, target regions for each miRNA family member were used to predict duplex structures with each miRNA with RNAhybrid. Here, simplified settings were used without consideration of canonical seeds (–f settings omitted). For motif analysis, enriched 6mer, 8mer, 10mer and 12mer motifs in target regions were determined with HOMER, using AGO-binding regions in the brain as the background^39^.

## Additional information

**Accession codes:** High-throughput sequencing data are available at NCBI GEO under the accession number GSE73059.

**How to cite this article:** Moore, M. J. *et al*. miRNA–target chimeras reveal miRNA 3′-end pairing as a major determinant of argonaute target specificity. *Nat. Commun.* 6:8864 doi: 10.1038/ncomms9864 (2015).

## Supplementary Material

Supplementary Information Supplementary Figures 1-10, Supplementary Tables 1-7 and Supplementary References

Supplementary Data 1 miRNA-target interactions in mouse brain.

Supplementary Data 2 Ago binding peaks in mouse brain, annotated with miRNA seed matches and overlapping chimeras.

Supplementary Data 3 Gene ontology (GO) enrichments for major brain miRNAs. FDR values for each miRNA in each category are shown (hypergeometric test). ‘n.s.' = not significant (FDR > 0.01).

Supplementary Data 4 miRNA-target interactions in human hepatoma (Huh7.5) cells.
