# Supplementary material for: miRNA–target chimeras reveal miRNA 3′-end pairing as a major determinant of Argonaute target specificity
Source: Nat Commun. 2015 Nov 25;6:8864. doi: 10.1038/ncomms9864 (PMC4674787; doi:10.1038/ncomms9864)
Supplement: Supplementary Information — Supplementary Figures 1-10, Supplementary Tables 1-7 and Supplementary References [file ncomms9864-s1.pdf]

Supplementary Figures

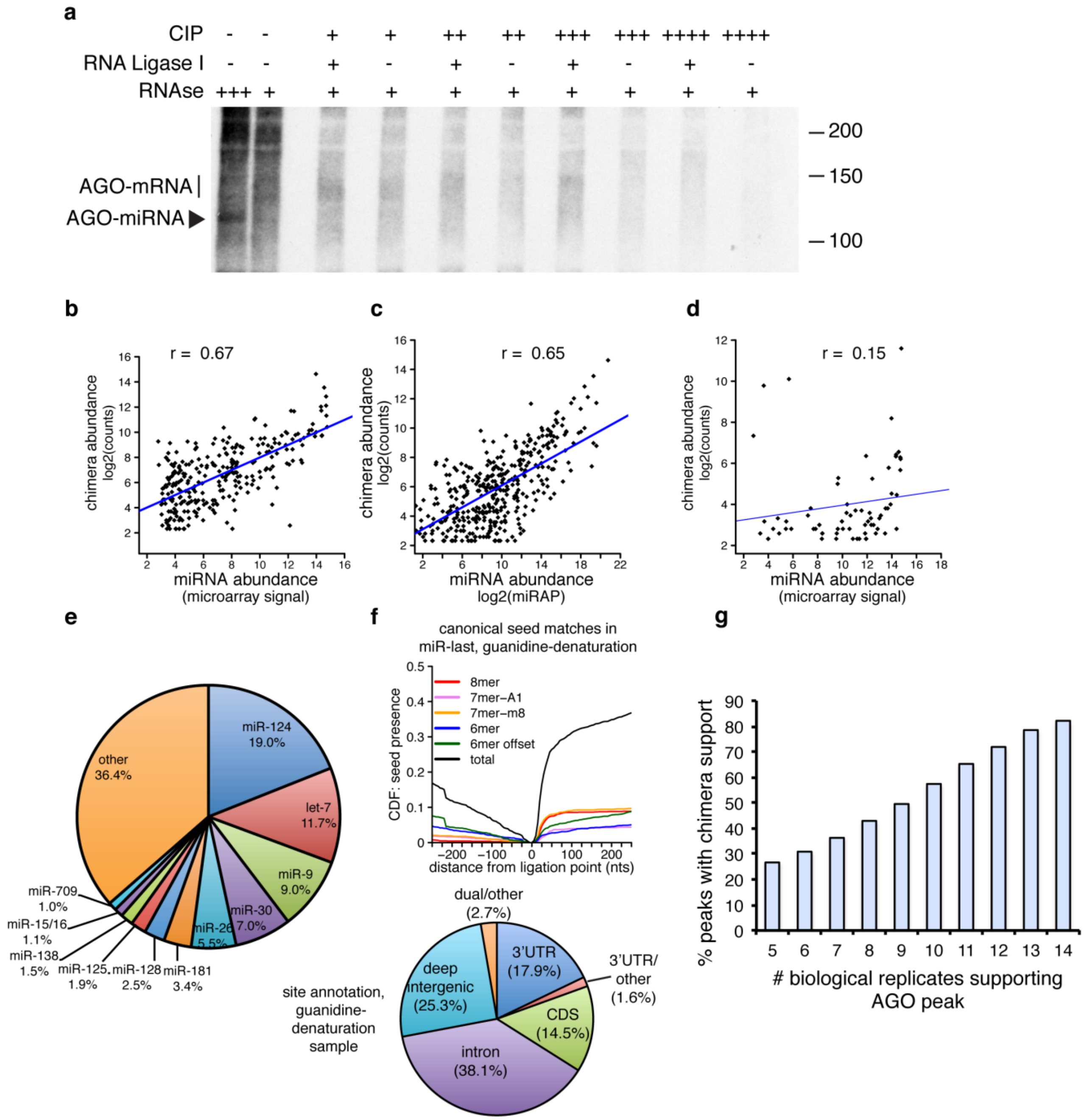

Supplementary Figure 1. Supporting data for CLEAR-CLIP (a) Purified AGO complexes from brain were labeled with PNK and ^{32}P-[illegible]-ATP, then incubated with (+) or without (-) T4 RNA ligase I as indicated. Complexes were subjected to varying levels of alkaline phosphatase (CIP) treatment to assess ligase-mediated protection from CIP de-phosphorylation. Sample treated with high levels of RNAse (+++) show the migration of AGO-miRNA complexes at ~110 kD[1,2] (b-c) Correlation plots of miR-first chimera abundance versus brain miRNA abundance measured by (b) miRNA tagging and affinity purification (miRAP) from cortex [3] and (c) microarray in whole brain (GEO accession GSE26271). (d) Correlation plots of miR-last chimeras versus miRNA abundance measured by microarray in whole brain. (f) CDF for canonical seed presence in samples processed with guanidine HCl denaturation. (g) The percentage of AGO binding peaks in brain with chimera support is plotted. AGO binding peaks are stratified by biologic complexity (BC), the number of independent biological replicates supporting that peak.

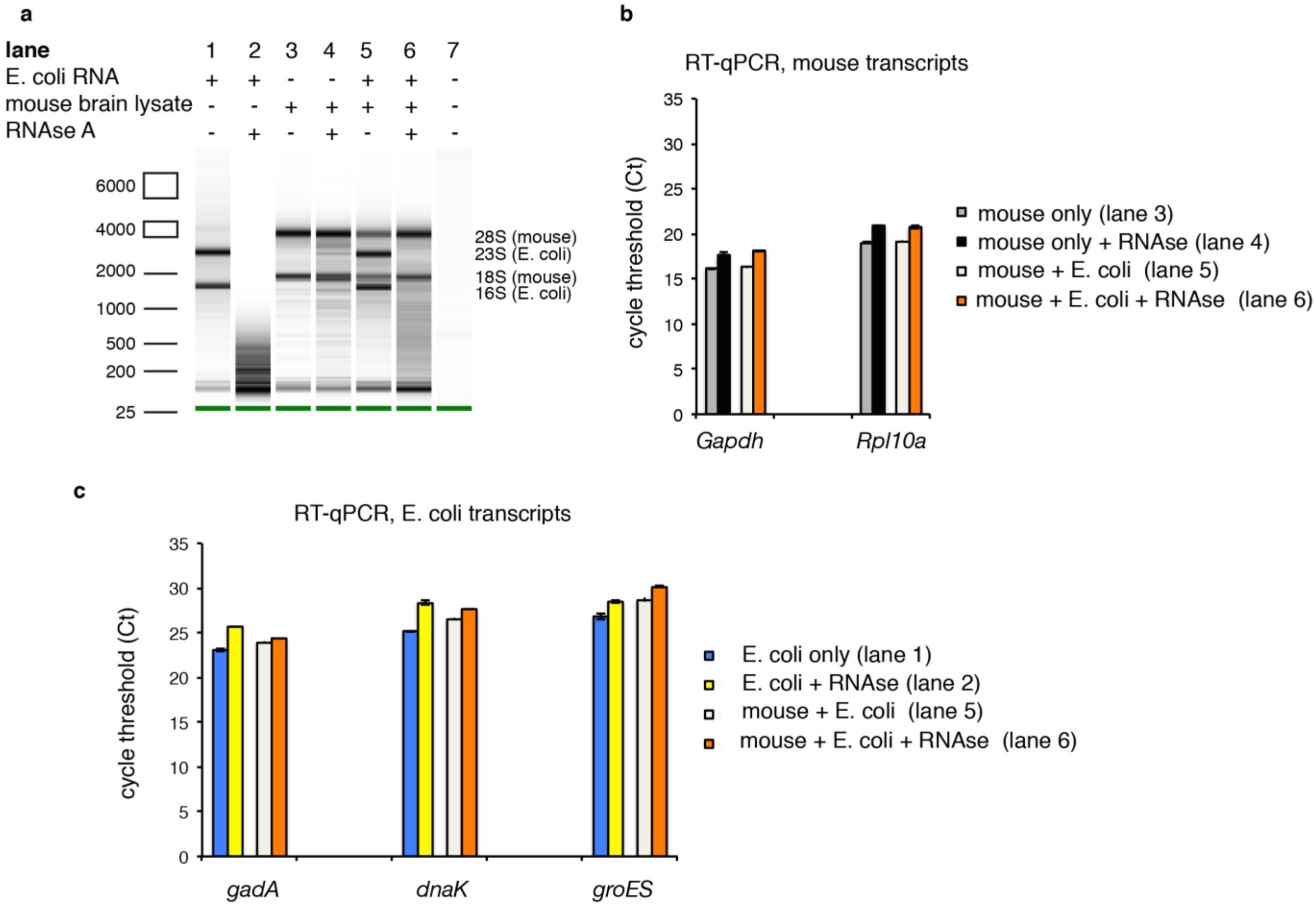

Supplementary Figure 2. E. coli RNA mixed into mouse brain lysates. (a) Bioanalyzer traces are shown for purified E. coli total RNA (lane 1), mouse brain lysate RNA (lane 3), and purified E. coli mixed into mouse brain lysate (lane 5), and for those samples treated with exogenous RNAse A as in CLEAR-CLIP (lanes 2, 4, and 6, respectively). After indicated treatments, RNA was extracted with Trizol LS. MW markers are indicated on the left, and the migration of rRNA species on the right. Note that E. coli RNA remains in tact in the absence of exogenous RNA in brain lysates (lane 5). RNAse treatment cleaves E. Coli RNA to fragments suitable for CLIP in lysis buffer alone (lane 2) and in mixed brain lysates (lane 6). Note that mouse rRNA fragments are very protected from RNAse (lanes 4 and 6), as mouse ribosomes remain in tact in brain lysate. E. coli rRNA is not similarly protected (lane 6) because the E. coli extraction procedure denatures ribosomes (see Methods). (b) Equal volume amounts of the indicated RNA samples from (a) were analyzed by RT-qPCR of mouse transcripts with 60-70 bp amplicons. Increased cycle threshold (Ct) values upon RNAse treatment reflect partial cleavage, consistent with generation of fragments suitable for CLEAR-CLIP. (c) RT-qPCR analysis of E. coli transcripts from indicated samples from (a) was performed with 60-70 bp amplicons. As for mouse, increased Ct values upon RNAse treatment reflect partial cleavage, consistent with generation of CLEAR-CLIP fragments. In the absence of exogenous RNAse, there is only a small amount of cleavage (compare lanes 1 and 5), consistent with low endogenous RNAse activity. Data in (b) and (c) show the means of three PCR measurements +/- SD. Data from one of two independent experiments with similar results are shown.

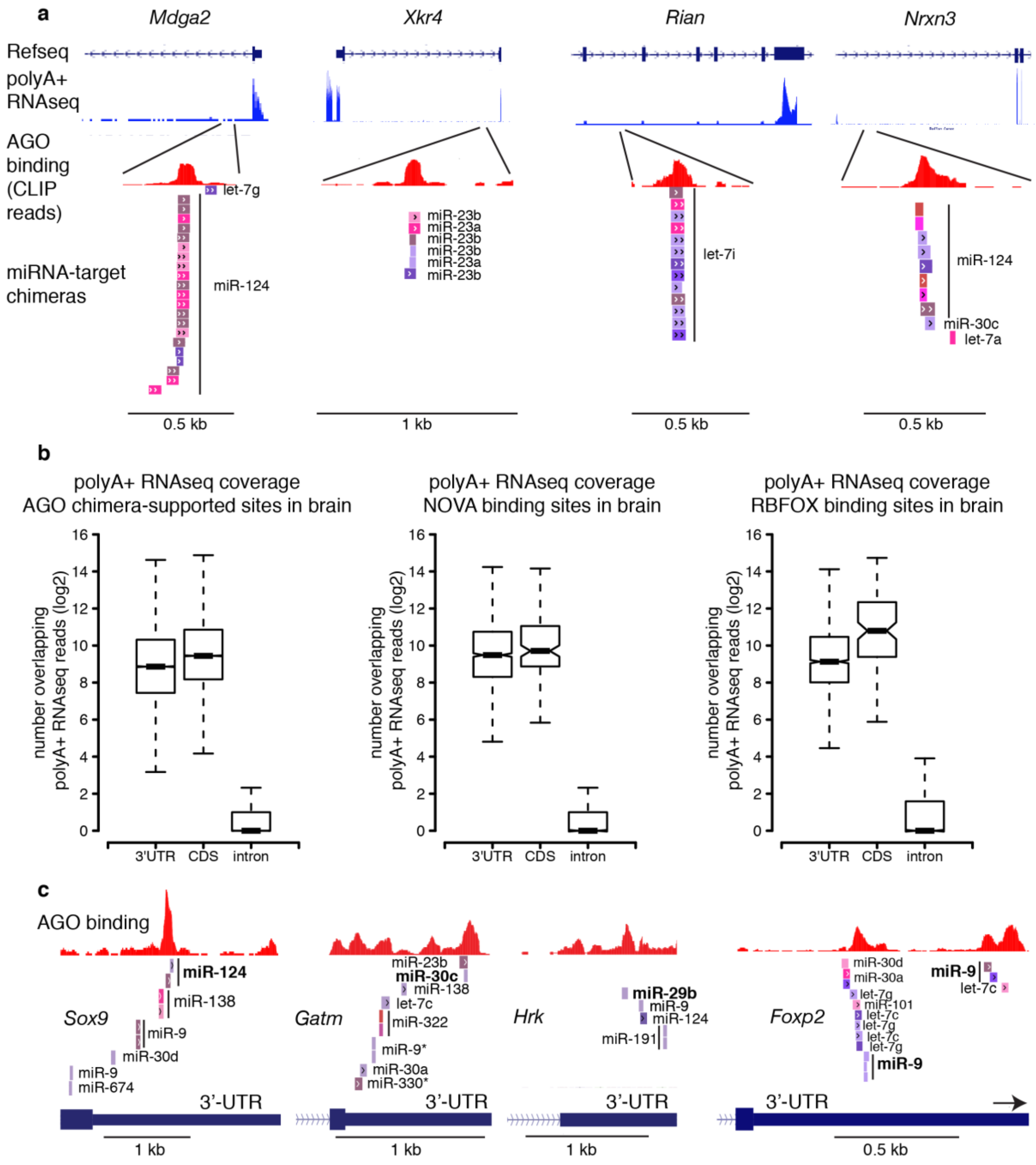

Supplementary Figure 3 CLEAR-CLIP identifies novel and previously known miRNA binding sites. (a) Examples of miRNA-dependent AGO binding in introns. Refseq tracks are shown along with polyA+ RNAseq data (blue) from age-matched mouse cortex in bedGraph format. AGO HITS-CLIP binding data in bedGraph format (red) in P13 cortex is shown, along with miRNA-target chimeras from CLEAR-CLIP. (b) PolyA+ RNAseq coverage was measured by counting the number of RNAseq reads from mouse cortex overlapping AGO chimera-supported sites in the indicated transcript regions (left panel). Coverage of intronic sites was significantly lower than exonic (p~0 vs. both CDS and 3'UTR site by two-tailed t-test), consistent with correct intronic annotation. For comparison, similar analyses were done for NOVA and RBFOX binding sites in brain, which also include exonic and intronic regions. (c) Examples of known miRNA regulation recovered by miRNA-target chimeras are shown, with previously reported interactions bolded[4-6]. Complete 3' UTRs are shown, except Foxp2, which continues downstream, indicated by arrow.

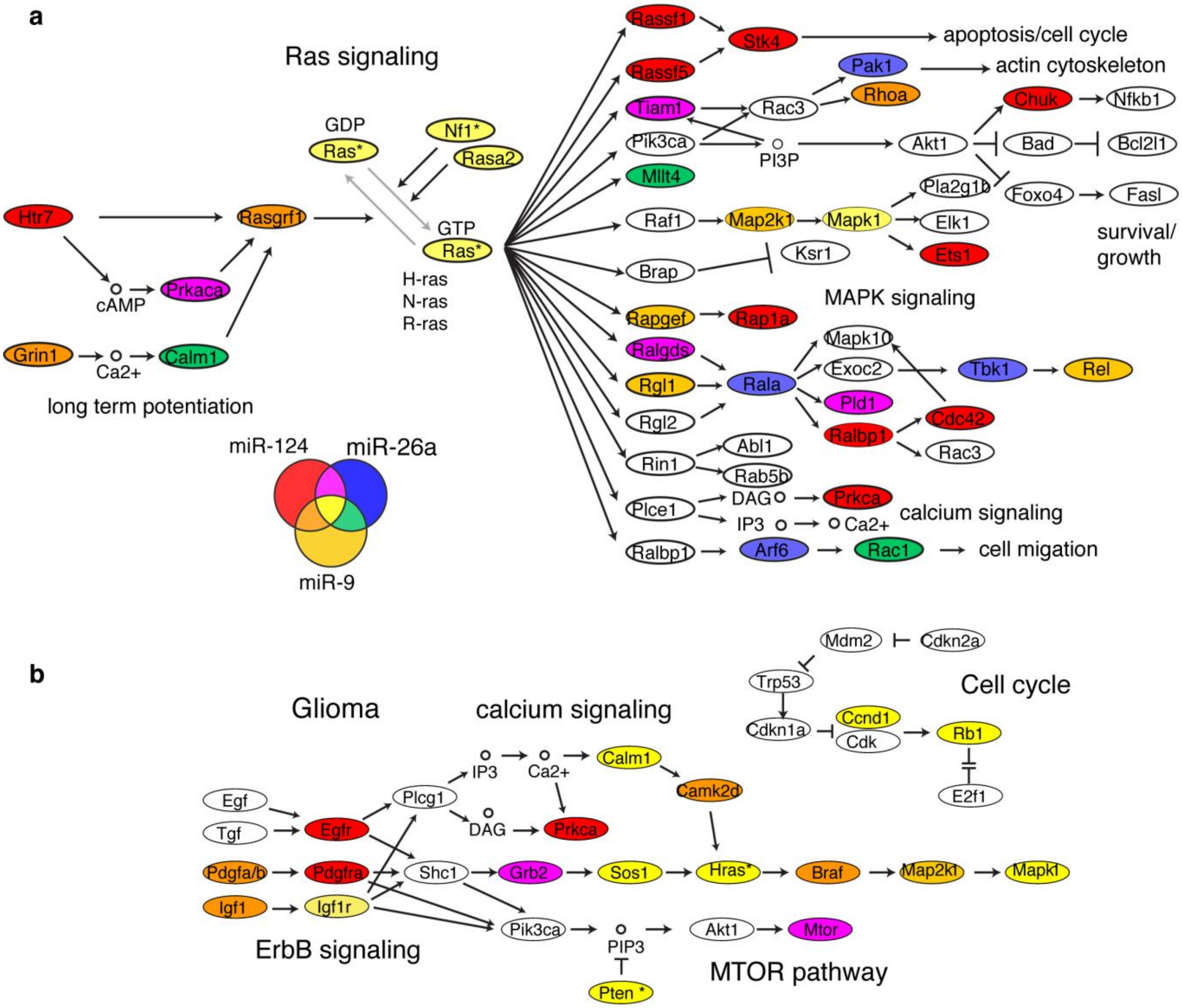

Supplementary Figure 4. Novel targets for brain miRNAs linked to glioma. (a) The KEGG Ras signaling pathway is shown with 3'UTR and CDS targets of targets of enriched miRNAs miR-124, miR-9, and miR-26a highlighted [7,8]. (b) The KEGG glioma pathway is shown with targets of indicated miRNAs. miR-9 and miR-26a had statistically significant enrichments for glioma pathway components ($p<0.05$, hypergeometric test, Bonferroni corrected), and miR-124 was nearly significant ($p=0.07$) [7]. Previously identified targets are labeled with an asterick (*)[9-11].

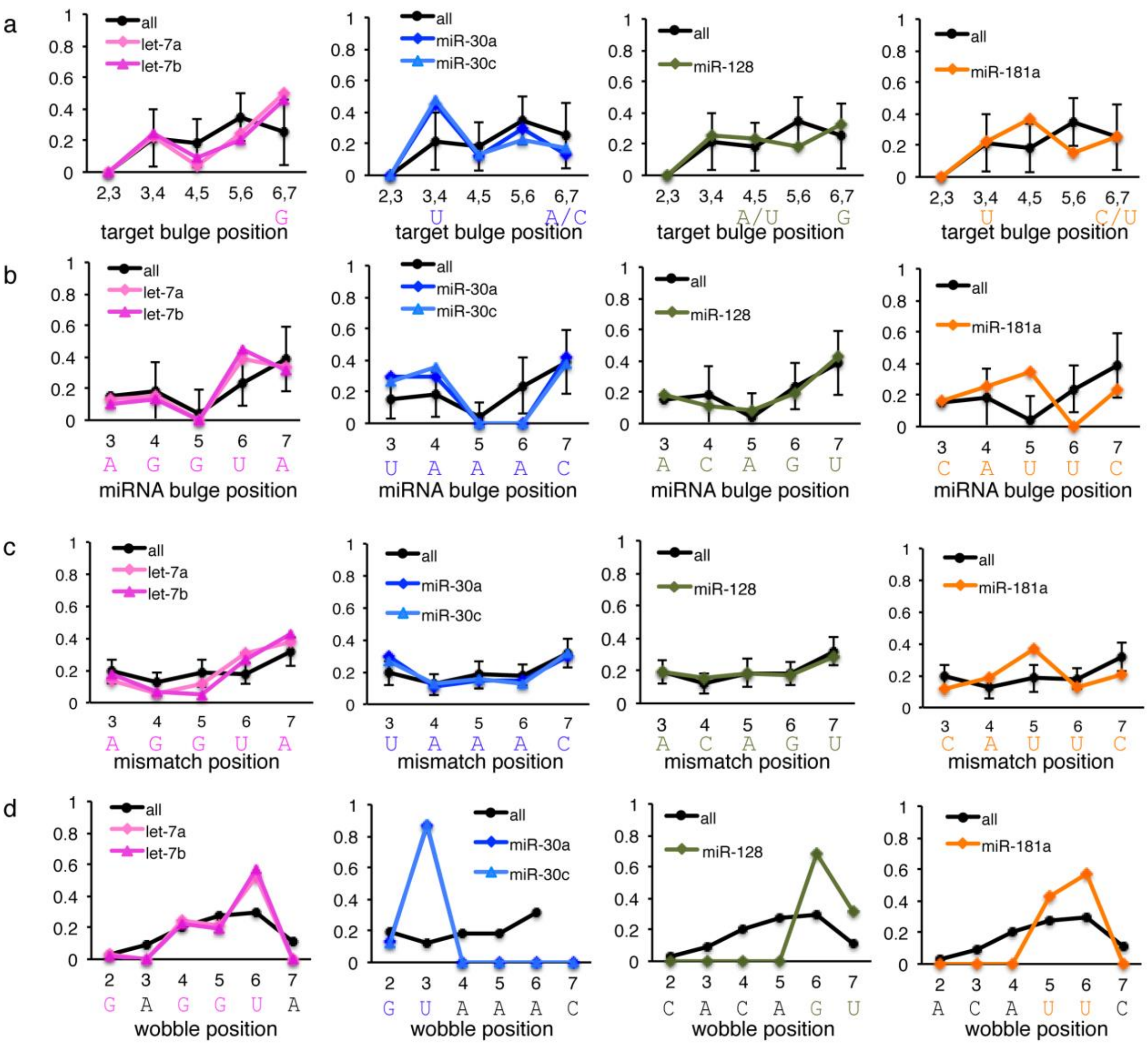

Supplementary Figure 5. miRNA-specific preferences for bulged nucleotides in seed region. (a) For sites with bulged target seed interactions, the fraction occurring at each position is plotted for representative miRNAs. Average values for all bulged miRNA sites are shown in black, +/- SD. Below the plots, the nucleotide(s) most often bulged out at specific sites is shown if there was a clear preference at that site (>50% of observed cases). (b) Plots as in (a) for sites with bulged miRNA nucleotides. miRNA nucleotides are shown at each position. (c) Plots as in (a) shown for non-Watson Crick mismatches in seed regions. (d) Plots showing positions of G-U wobble interactions in seed regions. The colored nucleotides below the plots indicate positions where G-U pairing is possible.

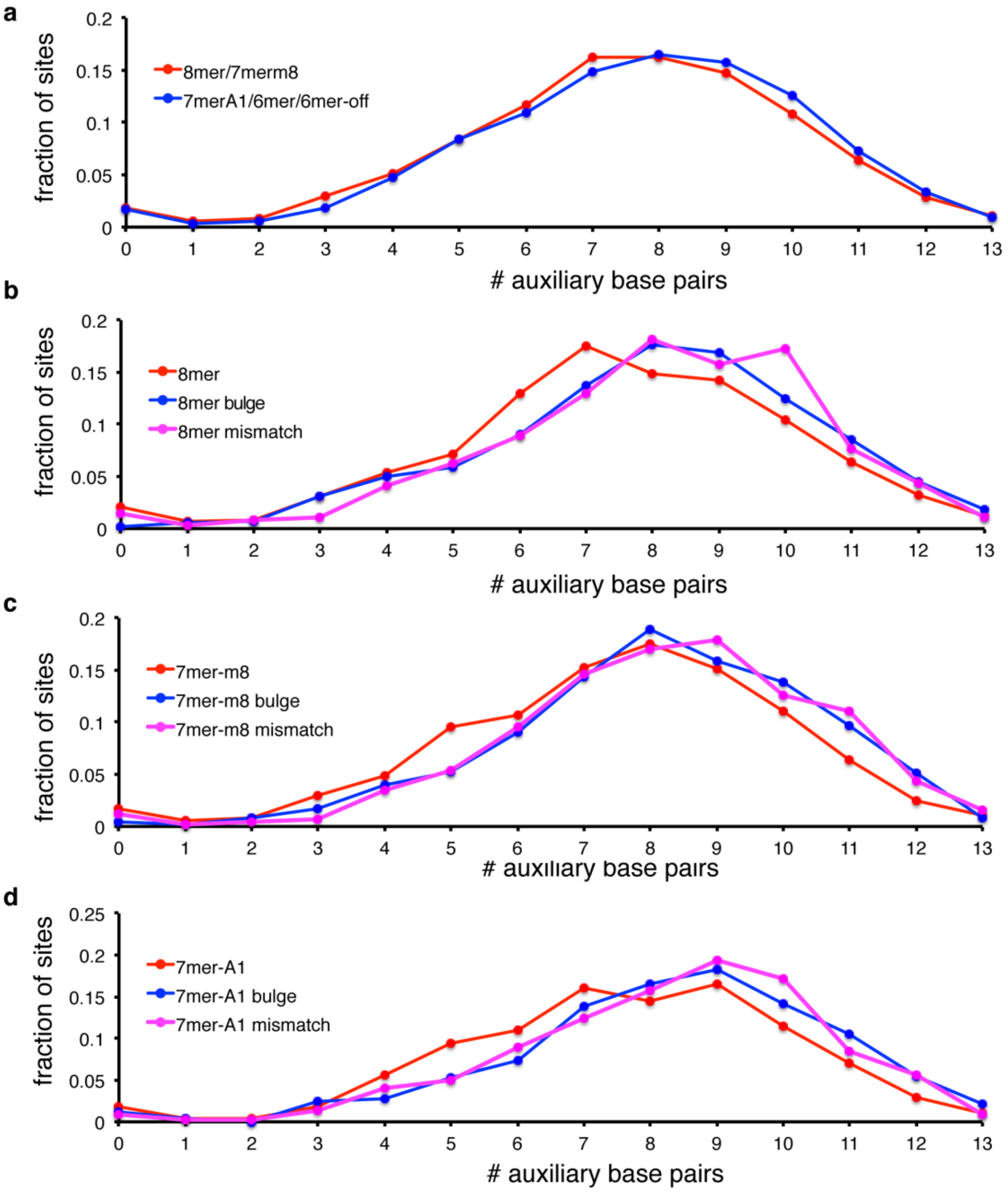

Supplementary Figure 6. Global evidence that imperfect seed matches are stabilized by 3' end pairing. (a) Numbers of auxiliary (non-seed) bases predicted to be engaged in complementary base pairing are plotted for canonical interactions with 7 (8mer/7mer-m8, red) or 6 (7mer-A1/6mer, blue) paired bases in the seed region. Sites with 6 paired seed bases had significantly more predicted auxiliary pairing (p=0.0018). (b) Plot as in (a) showing that bulged (blue, $p=2.2 \times 10^{-5}$) and mismatched (magenta, $p=6.0 \times 10^{-8}$) 8mer sites have significantly more auxiliary pairing than perfect 8mer matches (red). (c) Plot as in (b) for 7mer-m8 bulged ($p=7.4 \times 10^{-12}$) and mismatched ($p=1.9 \times 10^{-15}$) sites. (d) Plot as in (b) for 7mer-A1 bulged ($p=3.4 \times 10^{-9}$) and mismatched ($p=2.0 \times 10^{-11}$) sites. P-values were calculated with two-tailed t-tests.

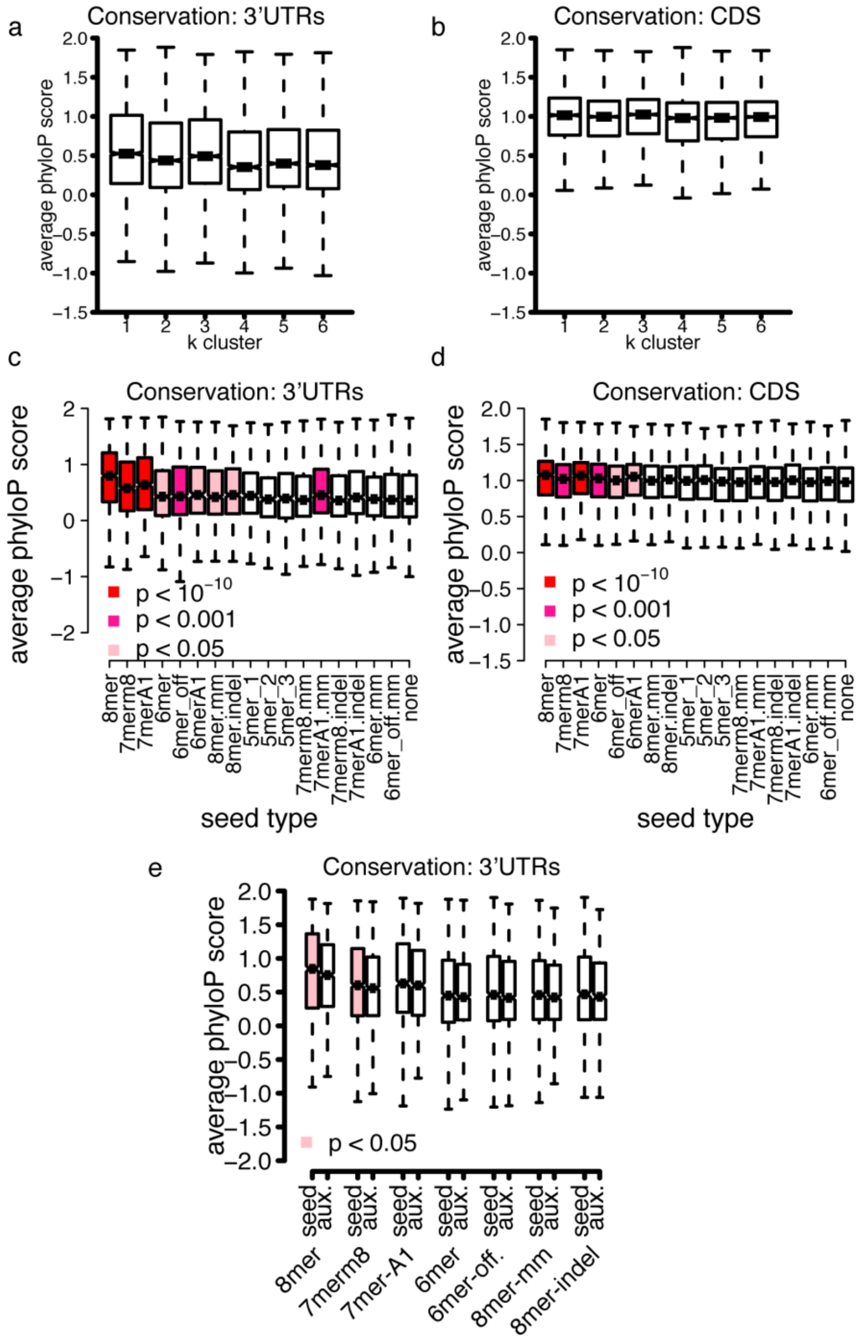

Supplementary Figure 7. Subsets of CLEAR-CLIP targets show enriched evolutionarily conservation. (a) Average phyloP conservation scores for 3'UTR and (b) CDS target regions is plotted for different k clusters. (c) Average phyloP conservation scores for 3'UTR and (d) CDS target regions with the indicated seed match variants are plotted. Significance was calculated with two-tailed t-tests (Bonferroni corrected) comparing different seed classes to regions lacking seed homology ("none"). (e) Interactions with the indicated seed homology were divided into seed and auxiliary regions based on predicted duplex structures. Average phyloP scores within those sub-regions are plotted for each set. Seed regions for 8mer interactions were more conserved than their auxiliary regions ($p < 0.05$, two-tailed t-test). Differences for other seed types were not significant.

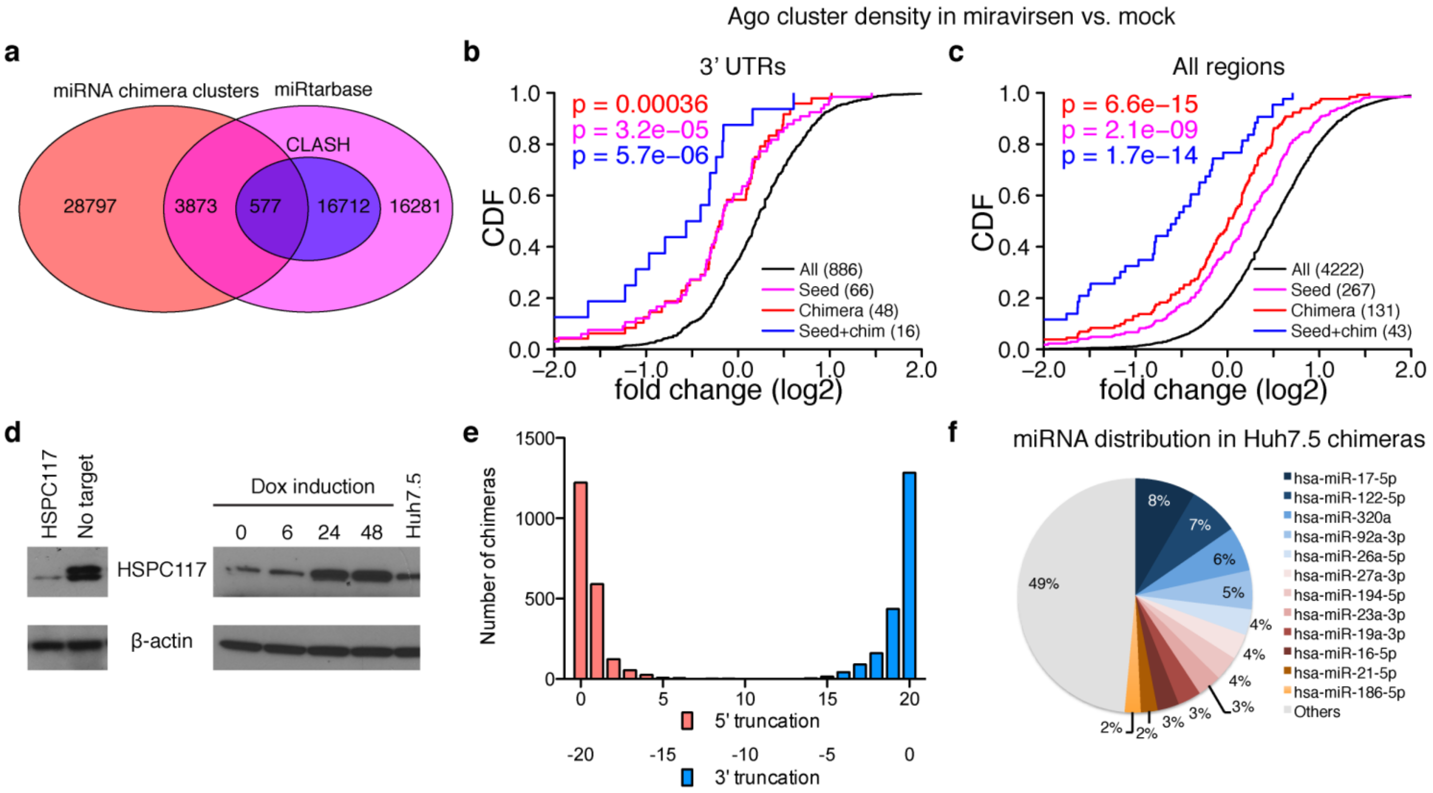

Supplementary Figure 8. Characterization of miRNA-target interactions in human hepatoma cells. (a) Venn diagram of overlap of miRNA-target interactions identified in Huh-7.5 cells with interactions annotated in miRtarbase. (b-c) CDF plots of miravirsen induced changes in AGO binding across 3'UTRs (b) or all regions (c) for sites identified by the presence of miR-122 7-8mer seeds, miR-122 chimeras, or the combination of both. P-values are shown from K-S tests comparing the indicated subsets to control (black) sets. (d) Western blots of knock-down (left) and doxycycline-induced over-expression 0-48 hrs post induction (right) of HSPC117. (e) Number of chimeras with indicated miRNA truncation lengths from the 5' or 3', as determined by Novoalign (Novocraft). (f) The distribution of miRNAs identified in chimeras in Huh-7.5 cells.

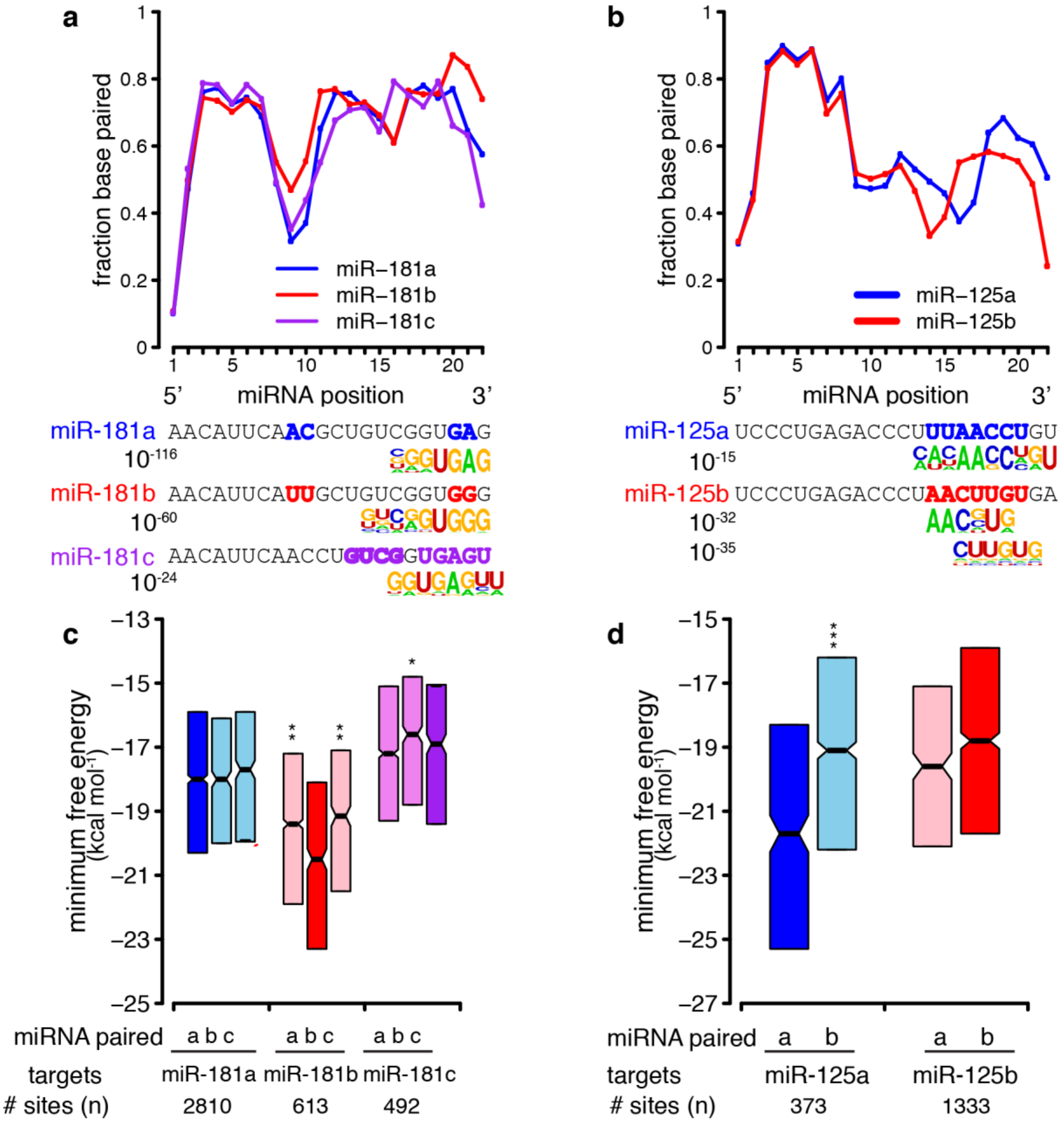

Supplementary Figure 9. Additional examples of miRNA family member specificity. (a-b) Base pairing profiles as in Fig. 7a-b are shown for (a) miR-181 and (b) miR-125 family members. Paralog-specific enriched motifs are shown below plots, with p-values (hypergeometric tests by HOMER) for enrichment over background (AGO binding regions in brain). (c-d) Pairwise duplex structure analyses as in Fig. 7c-d are shown for (c) miR-181 and (d) miR-125 family members. Predicted minimum free energy (MFE) is plotted, with boxplots representing interquartile (25-75) values (* = $p < 0.05$; ** = $p < 0.001$; *** = $p < 10\text{-}^{10}$, one-tailed t-test).

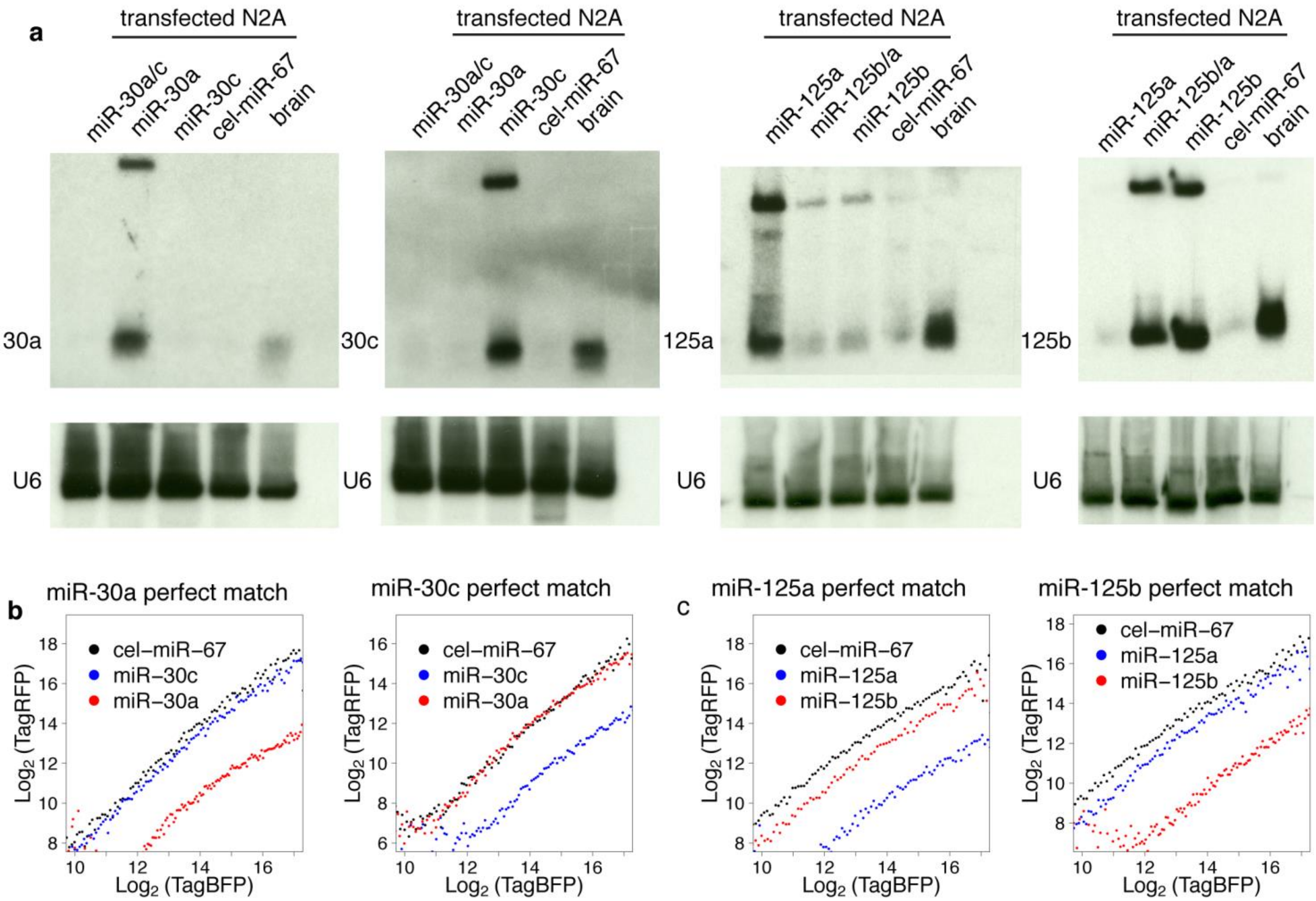

Supplementary Figure 10. Efficient miRNA expression from genomic fragments. (a) Northern blotting analysis of RNA from N2A cells transfected with plasmids expressing the indicated miRNAs and, for comparison, mouse cortex. Probes used for each membrane are indicated to the left of images. For miR-30a, plasmids expressing miR-30a from its endogenous locus (miR-30a) and inserted into the miR-30c locus (miR-30a/c) were tested, but only the endogenous locus showed robust expression. For miR-125b, expression from the endogenous (miR-125b) and miR-125a locus (miR-125b/a) were robust. U6 snRNA is shown as a loading control. (b-c) Log-log transformed correlation plots for tagRFP versus tagBFP for constructs with sites perfectly complementary to (b) miR-30 paralogs and (c) miR-125 paralogs.

Supplementary Tables

Supplementary Table 1. Read statistics and experimental details for P13 mouse cortex CLEAR-CLIP samples. Information for each sample includes: a description of RNAse A treatment (pre- or post-IP; see Methods); AGO-RNA complex molecular weight (MW) isolated from the autoradiogram; total non-chimeric CLIP reads mapped to mm9; unique non-chimeric read numbers after PCR duplicate collapse; mapped and unique read numbers for miR-last and miR-first chimeras; and miR-first chimeric reads as a fraction of unique CLIP reads. Index refers to primer indices used for multiplexing in the second PCR amplification step (see also Supplementary Table 7). For sequencing run, H1 and H2 were two different Hiseq 2500 single-end 100 nucleotide run, and M1-M3 were Miseq single-end 75 nucleotide runs.

CLEAR-CLIP samples	RNAse	complex MW	mapped CLIP reads	unique CLIP reads	mapped miR-last chimeras	unique miR-last chimeras	mapped miR-first chimeras	unique miR-first chimeras	chimera fraction	index	seq run
BR1	pre	150-180	875235	558790	2006	1108	19312	13048	0.023	ACCG	H1
BR2	pre	150-180	593142	179422	2733	462	9102	2659	0.015	CATG	H1
BR3	post	150-180	710554	381937	2063	432	14017	8558	0.022	TGCA	H1
BR6	post	130-150	3858045	1406126	12562	2117	189995	63753	0.045	TCAC	H1,M1
BR7	post	180-200	656152	301666	295	145	7696	3816	0.013	AGTG	M1
BR8	post	180-200	297089	235730	133	79	3817	2869	0.012	TACG	M1
BR10a	post	150-180	599990	367634	1659	829	11055	7436	0.020	CGTA	H1
BR10b	post	180-200	505927	254674	1818	710	7019	4221	0.017	GCAT	H2
BR11	post	150-180	759959	472388	2127	1002	14474	9984	0.021	CCGA	H1
BR12a	post	150-180	1068562	619777	3455	1490	19055	12348	0.020	GAGG	H1
BR12b	post	180-200	603237	266867	2332	800	9556	4728	0.018	AACC	H2
BR13	pre	130-150	171912	87886	69	42	2444	1363	0.016	ATGC	M2
BR14	pre	130-150	156773	98655	59	50	2504	1730	0.018	TAGC	M2
BR15	pre	130-150	473856	381595	142	219	9641	8046	0.021	CTAG	M3
BR16	pre	130-150	514853	454129	217	193	9498	8523	0.019	GATC	M3
BR17	pre	130-150	164776	99681	70	45	2759	1784	0.018	CGTA	M2
BR18	pre	130-150	163564	85500	68	43	2540	1312	0.015	GCAT	M2
BR19	pre	130-150	396886	294289	152	132	3431	2660	0.008	TGCA	M3
BR20	pre	130-150	460875	366855	150	130	6504	5442	0.015	ATGC	M3
**Minus Ligase Samples**	**RNAse**	**complex MW**	**mapped CLIP reads**	**unique CLIP reads**	**mapped miR-last chimeras**	**unique miR-last chimeras**	**mapped miR-first chimeras**	**unique miR-first chimeras**	**chimera fraction**	**index**	**seq run**
BR4	pre	150-180	810230	449580	1882	918	1829	1229	0.003	TCAC	H1
BR5	post	150-180	885101	655357	2311	1362	1825	1254	0.002	AGTG	H1
**Denatured Samples**	**RNAse**	**complex MW**	**mapped CLIP reads**	**unique non-chimeric reads**	**mapped miR-last chimeras**	**unique miR-last chimeras**	**mapped miR-first chimeras**	**unique miR-first chimeras**	**chimera fraction**	**index**	**seq run**
BR9	pre	130-150	177605	41127	7303	430	19534	2300	0.056	GATC	H2

Supplementary Table 2. Read statistics and experimental details for mixing experiments. Information for each sample includes: a description of RNAse A treatment (pre- or post-IP; see Methods); AGO-RNA complex molecular weight (MW) isolated from the autoradiogram; the composition of each sample (mouse brain alone; mouse brain plus *E. coli*; mouse brain plus *D. melanogaster*); unique non-chimeric read numbers mapped to mm9 after PCR duplicate collapse; unique non-chimeric read numbers mapped to the *E. coli* (or *D.* melanogaster) genome; unique chimeric read numbers mapped to mm9; and unique chimeric read numbers mapped to *E. coli* (or *D. melanogaster*)*.* Index refers to primer indices used for multiplexing in the second PCR amplification step (see also Supplementary Table 11). For sequencing run, M2, M3, and M4 were Miseq single-end 75 nucleotide runs

**Samples**	**RNAse**	**complex MW**	**composition**	**unique CLIP reads, mm9**	**unique CLIP reads, *E. coli***	***E. coli* fraction of CLIP reads**	**Unique miR-first chimeras, mouse**	**Unique miR-first chimeras, *E. coli***	**E. coli fraction of chimeric reads**	**index**	**seq run**
BR13	pre	130-150	mouse only	87886	828	0.009	1363	26	0.019	ATGC	M2
BR14	pre	130-150	mouse only	98655	761	0.007	1730	32	0.018	TACG	M2
BR15	pre	130-150	mouse only	383380	6110	0.016	8046	6	0.001	CTAG	M3
BR16	pre	130-150	mouse only	456116	6600	0.014	8523	9	0.001	GATC	M3
BR17	pre	130-150	mouse + *E. coli* (1:1)	99681	797	0.008	1784	38	0.021	CGTA	M2
BR18	pre	130-150	mouse + *E. coli* (1:1)	85500	764	0.009	1312	23	0.017	GCAT	M2
BR19	pre	130-150	mouse + *E. coli* (1:6)	295785	7164	0.023	2660	146	0.052	TGCA	M3
BR20	pre	130-150	mouse + *E. coli* (1:6)	368618	9134	0.024	5442	301	0.052	ATGC	M3
**Samples**	**RNAse**	**complex MW**	**composition**	**unique CLIP reads, mm9**	**unique CLIP reads, dm3**	**fly fraction of CLIP reads**	**Unique miR-first chimeras, mouse**	**Unique miR-first chimeras,fly**	**fly fraction of chimeric reads**	**index**	**seq run**
BR21	pre	130-150	mouse only	465747	949	0.002	13510	100	0.007	GATC	M4
BR22	pre	130-150	mouse + fly (1:1)	530286	2683	0.005	14437	470	0.032	CGTA	M4
BR23	pre	130-150	mouse + fly (1:1)	470904	2365	0.005	12303	343	0.027	GCAT	M4

Supplementary Table 3. Enrichments of brain miRNAs in interaction classes defined by k-means clustering of duplex structures for mouse brain miRNAs. Enrichment values for miRNAs with >50 identified interactions in six interaction classes defined in mouse brain. Values are –log(p-value), calculated by Fisher's exact test. Positive values shaded in red indicate overrepresentation in a given class (enrichment), and negative values shaded in blue indicate underrepresentation (depletion).

	**k group**					
**microRNA**	**1**	**2**	**3**	**4**	**5**	**6**
mmu-let-7a	90.92	5.27	-77.19	-57.78	-0.31	0.89
mmu-let-7b	-153.36	19.80	-40.08	-39.84	4.03	124.86
mmu-let-7c	-102.30	0.73	-64.49	-53.30	49.10	113.49
mmu-let-7d	20.22	1.44	-12.91	-15.47	-7.79	6.66
mmu-let-7d*	1.27	0.13	-0.55	2.77	-3.05	-0.20
mmu-let-7e	25.81	0.96	-29.78	-14.09	-0.21	2.15
mmu-let-7f	19.97	1.72	-2.60	-25.74	-2.59	0.35
mmu-let-7g	1.51	3.19	-1.40	-15.61	-1.12	2.84
mmu-let-7i	-34.88	16.12	-0.34	-8.88	-6.99	20.27
mmu-let-7i*	-0.80	-1.19	0.00	0.00	-1.33	5.70
mmu-miR-100	-0.42	-2.49	-0.65	1.03	4.99	-0.28
mmu-miR-101a	3.81	5.84	-6.89	-0.14	0.00	-4.46
mmu-miR-101b	-0.39	22.53	-1.67	-0.09	-3.96	-5.85
mmu-miR-103	-16.19	15.61	0.98	-4.48	-36.10	16.53
mmu-miR-106a	-5.68	-5.46	24.36	0.99	-4.62	-0.29
mmu-miR-106b	0.20	8.99	-0.19	-0.18	-5.67	-1.51
mmu-miR-107	-4.14	29.22	0.10	-7.95	-33.81	3.91
mmu-miR-1186	-1.40	0.48	9.36	-0.43	-1.44	-3.13
mmu-miR-1186b	10.09	4.53	-9.83	0.09	-2.75	-2.82
mmu-miR-1196	2.64	-12.42	-20.23	96.10	-0.21	-20.45
mmu-miR-1198-5p	-1.29	-0.05	0.19	0.00	-1.28	2.82
mmu-miR-1224	-5.34	19.68	-11.92	-3.38	-2.49	3.12
mmu-miR-124	244.81	2.67	-244.81	-139.43	244.81	-173.97
mmu-miR-1249	6.66	-0.93	-0.26	-2.28	-0.70	0.23
mmu-miR-125a-5p	-5.65	-30.45	10.06	-2.67	29.01	-1.83
mmu-miR-125b-2-3p	-0.31	1.65	-0.29	3.77	-2.50	-0.73
mmu-miR-125b-5p	-2.04	-1.71	18.40	-4.14	-0.39	-0.26
mmu-miR-126-3p	1.20	-18.16	-3.42	-0.07	20.05	-0.12
mmu-miR-126-5p	2.18	3.14	3.54	4.18	-12.01	-11.71
mmu-miR-127	-16.44	-0.94	4.87	-1.67	-4.85	16.19
mmu-miR-127*	-0.07	3.79	0.38	-0.04	-4.63	-0.16
mmu-miR-128	79.40	43.02	1.65	-5.04	-128.30	-42.15

mmu-miR-129-1-3p	15.62	-2.25	-0.15	-0.94	0.20	-6.23
mmu-miR-129-2-3p	6.35	-6.67	0.22	-1.35	10.45	-11.90
mmu-miR-129-5p	0.27	14.09	-5.33	3.16	-25.76	0.42
mmu-miR-1298	0.00	0.29	-0.41	-0.18	-0.22	0.69
mmu-miR-130a	-28.25	0.24	5.43	-10.88	6.52	1.15
mmu-miR-132	-3.05	0.30	2.28	-0.63	0.39	-0.19
mmu-miR-134	-4.44	-2.31	0.00	-0.41	0.82	6.06
mmu-miR-135a	-1.49	-7.81	12.56	-2.42	-1.15	2.38
mmu-miR-135b	0.00	-5.48	7.09	-2.09	-0.49	1.13
mmu-miR-136	-3.29	-4.95	-0.13	6.32	0.28	1.88
mmu-miR-137	-0.62	-4.02	2.49	3.63	2.78	-4.15
mmu-miR-138	-48.31	-25.13	0.29	-21.49	46.96	27.80
mmu-miR-139-3p	-1.69	1.36	-2.34	-0.28	0.13	1.81
mmu-miR-139-5p	-6.48	-0.55	15.01	6.09	-16.01	0.24
mmu-miR-141	-0.62	-1.70	-0.47	2.39	2.27	-0.21
mmu-miR-142-3p	-0.51	-4.40	1.11	0.14	0.16	1.58
mmu-miR-143	-0.29	14.18	-0.09	-1.56	-1.07	-4.66
mmu-miR-144	7.49	0.16	-0.41	-0.85	-2.02	-0.82
mmu-miR-145	3.71	-7.13	0.26	-2.11	-0.26	2.76
mmu-miR-146a	-7.24	-0.65	15.47	0.39	-0.72	-2.27
mmu-miR-146b	-2.38	-2.19	8.75	-0.30	0.00	-0.20
mmu-miR-149	3.22	-0.95	-2.16	-10.57	-0.23	7.29
mmu-miR-150	-0.72	0.00	1.69	0.31	0.00	-0.57
mmu-miR-151-3p	-0.94	2.83	-8.32	0.00	1.38	0.32
mmu-miR-153	-1.80	12.13	20.32	-6.67	-2.80	-18.56
mmu-miR-154	-0.41	1.03	0.21	-2.15	-2.56	2.55
mmu-miR-15a	-2.92	-6.76	41.29	-1.03	-19.49	0.44
mmu-miR-15b	1.98	1.18	-0.11	-0.33	-1.78	-0.43
mmu-miR-16	-15.41	-41.57	13.66	-9.79	74.03	-4.09
mmu-miR-17	-9.36	-5.66	24.50	0.95	-7.79	1.28
mmu-miR-181a	-110.81	-31.78	1.02	74.33	10.05	4.40
mmu-miR-181b	-36.22	-40.85	-1.33	21.07	6.96	15.08
mmu-miR-181c	-13.69	-1.19	7.49	6.66	-6.46	1.82
mmu-miR-181d	-24.82	-23.39	0.12	12.44	2.90	8.22
mmu-miR-182	-0.14	-0.91	-0.39	0.00	0.29	1.78
mmu-miR-1839-5p	-1.53	1.38	0.74	-1.06	-3.78	2.00
mmu-miR-185	1.43	-0.18	-5.34	-1.40	0.54	2.52
mmu-miR-186	-16.63	-9.69	8.46	2.77	11.92	-3.57
mmu-miR-187	-4.29	1.03	-0.36	-1.52	-3.96	9.56
mmu-miR-188-5p	-2.24	0.00	0.26	7.11	-7.09	0.73
mmu-miR-18a	0.00	1.79	0.16	-1.62	-3.68	1.06

mmu-miR-190	-0.94	-7.39	-0.28	-0.23	4.46	2.51
mmu-miR-191	-7.99	-12.04	8.50	6.50	1.14	-1.15
mmu-miR-193b	-0.81	-2.54	10.37	-0.28	-2.91	0.12
mmu-miR-194	-1.41	-2.37	4.48	1.02	0.00	-0.33
mmu-miR-195	-0.24	-0.86	0.18	-3.02	11.51	-2.86
mmu-miR-1983	2.62	0.27	-1.20	0.63	-2.70	0.09
mmu-miR-19a	5.79	-6.67	3.74	-1.92	0.00	-0.95
mmu-miR-19b	13.82	-15.67	3.72	-0.82	0.38	-4.26
mmu-miR-200a	-0.36	0.65	-1.53	0.34	2.27	-1.05
mmu-miR-200b	2.79	-0.34	-2.87	0.13	3.14	-2.21
mmu-miR-200c	-2.91	-4.71	-2.24	5.67	5.93	0.04
mmu-miR-204	-0.24	-3.92	-0.94	0.36	0.22	4.75
mmu-miR-20a	-18.58	-7.40	49.80	0.12	-12.05	0.73
mmu-miR-21	-3.35	0.00	2.19	-1.08	-0.19	1.39
mmu-miR-211	-0.23	-0.90	-0.08	0.83	0.17	0.51
mmu-miR-212-3p	-3.81	0.20	0.06	0.00	0.21	0.91
mmu-miR-218	10.37	-0.57	0.58	-5.01	-4.14	0.12
mmu-miR-219-3p	2.13	0.00	-1.29	0.30	-0.06	-0.12
mmu-miR-219-5p	94.21	-37.61	30.63	-2.55	-10.12	-56.43
mmu-miR-22	2.06	-0.49	-0.61	-3.00	0.00	1.17
mmu-miR-221	-13.24	5.77	0.21	-0.93	-2.69	3.70
mmu-miR-222	-12.30	1.51	-4.81	0.06	4.03	2.39
mmu-miR-23a	65.98	0.00	-3.99	1.15	-9.01	-23.19
mmu-miR-23b	79.48	1.83	-9.29	-0.12	-16.51	-18.70
mmu-miR-24	4.62	6.31	2.78	-16.76	-20.74	0.08
mmu-miR-26a	-93.46	-51.87	64.19	40.66	3.31	-0.02
mmu-miR-26b	-2.10	-0.25	5.94	1.32	-0.92	-1.10
mmu-miR-27a	0.87	13.36	-1.78	-0.45	-5.21	-1.69
mmu-miR-27b	-0.29	23.05	-3.19	-1.10	-10.15	-0.09
mmu-miR-296-3p	1.74	8.15	-3.63	-1.01	-2.99	-0.62
mmu-miR-296-5p	1.51	4.07	-0.61	-1.71	-0.21	-2.41
mmu-miR-298	4.05	0.00	-3.39	-2.16	0.00	0.85
mmu-miR-29a	-2.26	0.86	-0.05	-5.41	3.14	0.36
mmu-miR-29b	-4.01	-0.82	2.32	-2.33	0.85	1.81
mmu-miR-29c	-1.16	1.11	0.36	-3.08	2.12	-0.54
mmu-miR-300	2.54	9.99	-1.54	1.82	-26.71	-0.13
mmu-miR-301a	1.52	0.97	0.05	-1.19	-4.62	0.52
mmu-miR-301b	-0.34	3.81	-1.07	-1.37	0.00	0.00
mmu-miR-3059	-0.09	-3.17	-1.77	0.25	1.38	2.86
mmu-miR-3068	1.23	3.02	-0.65	-1.38	-1.44	-0.20
mmu-miR-3085-3p	1.05	1.00	1.68	-0.76	-1.44	-1.97

mmu-miR-3099	-30.26	-5.82	2.28	-8.45	0.13	32.08
mmu-miR-30a	-34.74	9.37	37.50	69.18	-31.83	-47.68
mmu-miR-30b	-0.98	-15.06	14.17	25.92	-9.72	-1.17
mmu-miR-30c	-0.19	-86.48	27.88	114.82	-13.16	-6.60
mmu-miR-30d	-11.86	1.42	49.55	49.74	-20.67	-63.97
mmu-miR-30e	-22.69	7.83	16.79	43.06	-16.48	-27.50
mmu-miR-31	-0.66	1.23	-1.53	-1.79	1.36	0.36
mmu-miR-32	-1.53	-1.08	3.28	0.00	0.35	-0.11
mmu-miR-320	-19.39	6.09	0.04	7.61	-3.99	0.21
mmu-miR-322	-3.38	-2.29	8.42	-4.03	-0.10	1.18
mmu-miR-323-3p	0.49	0.00	-1.87	2.26	0.12	-0.47
mmu-miR-324-5p	-2.54	-3.55	3.78	-1.47	3.74	-0.04
mmu-miR-326	0.52	0.84	1.94	-0.78	-2.59	-0.55
mmu-miR-328	2.77	0.15	0.62	-2.62	-2.67	0.21
mmu-miR-329	9.33	-0.06	-2.18	0.00	-1.72	-0.87
mmu-miR-33	0.44	19.53	-3.52	-3.16	0.04	-6.75
mmu-miR-330	-3.62	-2.31	4.17	-1.95	-0.69	5.24
mmu-miR-330*	-1.42	-1.13	0.26	0.35	0.25	1.09
mmu-miR-331-3p	12.57	0.40	-0.22	-0.21	-3.64	-5.07
mmu-miR-335-3p	0.00	13.92	0.00	-0.22	-6.43	-3.86
mmu-miR-337-3p	-1.54	0.20	3.33	0.18	0.62	-4.11
mmu-miR-338-3p	0.30	0.00	0.12	-0.16	0.28	-0.25
mmu-miR-339-5p	-2.03	0.59	0.31	-0.18	0.00	0.36
mmu-miR-340-5p	0.17	6.58	0.00	2.56	-6.80	-4.18
mmu-miR-342-3p	-3.02	-7.80	52.85	-2.36	-7.84	-1.51
mmu-miR-344	-0.69	-0.47	6.88	-0.56	-2.03	0.00
mmu-miR-345-5p	-0.41	0.11	1.26	0.27	-1.11	0.05
mmu-miR-3470a	-16.82	-3.89	-0.43	-1.34	-21.11	60.54
mmu-miR-3470b	-23.08	0.30	-2.02	0.27	-34.53	51.89
mmu-miR-3473	61.10	-26.78	-45.05	-9.35	-36.64	50.81
mmu-miR-350	5.72	2.33	-2.43	-2.53	-0.94	-0.92
mmu-miR-351	-2.35	-3.24	2.12	-1.71	1.70	1.14
mmu-miR-361	-6.57	8.96	3.46	0.35	-4.98	-2.06
mmu-miR-365	0.49	1.61	0.85	-0.09	-2.25	-1.17
mmu-miR-369-3p	1.71	0.10	-4.24	4.94	-0.61	-0.82
mmu-miR-370	-1.43	-2.26	-2.38	-1.39	13.64	0.22
mmu-miR-374	0.45	-1.28	1.85	2.20	-0.41	-2.12
mmu-miR-374c	2.39	-1.20	-0.39	5.18	-1.00	-1.46
mmu-miR-376a	0.70	-0.08	4.45	0.90	-5.46	-1.64
mmu-miR-376b	9.17	0.10	1.72	2.98	-14.05	-5.92
mmu-miR-378	0.84	0.00	-2.01	-0.61	0.53	0.47

mmu-miR-379	-0.31	-0.26	0.35	-4.14	1.52	0.55
mmu-miR-379*	7.64	1.29	-2.59	-0.35	-2.15	-1.10
mmu-miR-380-3p	4.85	1.41	-2.42	-1.64	-0.65	-0.32
mmu-miR-381	-1.47	-1.88	-0.43	9.52	-2.38	1.69
mmu-miR-382	2.07	2.30	-1.66	-2.63	-10.36	4.11
mmu-miR-383	-0.88	0.00	-2.40	-0.43	0.81	2.71
mmu-miR-384-3p	1.30	1.69	0.73	-0.08	-1.72	-2.41
mmu-miR-384-5p	-0.50	0.61	1.37	0.37	-2.82	0.00
mmu-miR-409-3p	3.06	-0.36	0.32	-0.33	0.05	-1.66
mmu-miR-410	-6.35	1.67	0.00	35.48	-16.39	-2.09
mmu-miR-411	3.72	2.21	0.57	-0.67	-1.69	-5.64
mmu-miR-411*	8.55	0.65	-2.50	0.08	-1.85	-2.12
mmu-miR-429	-0.47	-0.81	-5.84	4.33	13.24	-5.89
mmu-miR-431	2.64	0.58	-0.85	-2.25	0.00	-0.26
mmu-miR-431*	-0.53	4.74	-1.32	-0.33	-3.22	1.13
mmu-miR-433	-11.34	0.31	-14.66	0.00	2.78	13.54
mmu-miR-434-3p	0.43	-5.28	1.01	15.99	-2.32	-1.50
mmu-miR-434-5p	1.13	-0.14	-1.03	0.47	-0.23	0.21
mmu-miR-451	-0.11	-0.69	0.88	3.14	0.17	-2.36
mmu-miR-484	0.83	21.97	-6.20	-2.22	-9.51	-0.32
mmu-miR-487b	0.14	0.75	-3.23	1.66	-1.60	0.71
mmu-miR-488	-1.32	6.82	-2.27	0.62	-0.68	-0.39
mmu-miR-495	3.51	-0.58	-5.58	7.15	-0.86	-0.62
mmu-miR-496	7.62	-0.28	0.89	-1.55	-1.58	-2.26
mmu-miR-497	0.64	0.29	1.16	-0.97	-3.39	0.35
mmu-miR-500	-4.28	0.40	-2.06	0.05	-1.35	8.57
mmu-miR-541	-30.55	-0.22	-0.30	-4.80	1.81	19.40
mmu-miR-544-3p	0.50	-1.46	1.63	0.06	-0.73	0.09
mmu-miR-598	0.00	-0.31	-0.49	0.00	0.00	1.37
mmu-miR-652	-3.57	3.08	6.84	-0.89	-5.84	-0.28
mmu-miR-664	-0.16	0.41	0.58	-0.32	0.00	-0.30
mmu-miR-665	0.89	2.48	-0.17	-0.84	-5.56	0.43
mmu-miR-666-3p	-1.68	0.06	-0.39	-0.28	-0.72	4.47
mmu-miR-667	-0.44	1.27	1.57	0.06	-2.85	-0.18
mmu-miR-671-5p	-7.12	-3.08	-1.37	-1.72	-3.14	31.83
mmu-miR-673-5p	-0.43	-0.36	0.50	-0.08	0.74	0.00
mmu-miR-674	-3.33	6.46	-3.45	-1.20	0.00	0.83
mmu-miR-690	7.24	1.67	-4.28	1.63	-0.99	-6.46
mmu-miR-700*	-0.75	0.21	1.52	-0.43	-1.30	0.59
mmu-miR-706	93.85	-14.34	-32.18	59.93	-38.30	-46.73
mmu-miR-708	-20.01	-27.32	52.84	-7.03	-0.28	2.64

mmu-miR-709	81.41	2.62	-56.31	-12.15	-2.14	-3.62
mmu-miR-744	5.37	-1.14	-3.54	-2.10	6.58	-2.99
mmu-miR-770-3p	-4.00	-0.46	4.21	-3.53	-2.85	5.31
mmu-miR-770-5p	-4.09	9.83	-0.14	-0.61	-7.29	1.32
mmu-miR-7a	-2.68	0.50	-1.37	-0.31	1.51	1.06
mmu-miR-7b	-0.81	-0.09	-1.48	-0.62	2.44	1.13
mmu-miR-874	0.00	3.41	0.00	-0.19	-4.34	0.17
mmu-miR-877	-0.64	-0.18	0.00	0.00	-0.82	3.14
mmu-miR-879	1.85	-1.02	0.60	-0.68	1.13	-1.59
mmu-miR-9	-109.24	4.32	129.48	8.65	-177.81	16.41
mmu-miR-9*	60.77	-9.12	-0.40	0.04	-1.68	-14.46
mmu-miR-92a	-2.14	8.66	-1.26	1.05	-2.94	-0.30
mmu-miR-92b	-4.64	0.97	-0.82	4.42	-3.41	2.76
mmu-miR-93	-7.87	-4.07	4.61	1.10	-4.33	8.30
mmu-miR-98	4.99	-0.38	0.00	-2.23	-0.22	-0.36
mmu-miR-99a	-1.91	-0.25	-0.55	0.86	-0.03	2.24
mmu-miR-99b	-1.63	-0.94	5.14	0.86	-1.46	0.00

Supplementary Table 4. Read statistics and experimental details for Huh7.5 AGO HITS-CLIP and CLEAR-CLIP (following page). Information for each sample includes: a description of RNAse treatment (see Methods); the presence of endogenous HSPC117 ligase, where (+) represents endogenous levels, (-) knock-down, and (++) over-expression as shown in Figure 5e and Supplementary Figure 6e; the addition (+) or absence (-) of T4 RNA ligase I; AGO-RNA complex molecular weight (MW) isolated from the autoradiogram; total non-chimeric CLIP reads mapped to hg18; unique non-chimeric read numbers after PCR duplicate collapse; mapped and unique read numbers for miR-last and miR-first chimeras; and miR-first chimeric reads as a fraction of unique CLIP reads. Index refers to primer indices used for multiplexing in the second PCR amplification step (see also Supplementary Table 10). Sequencing runs for each samples are indicated. For sequencing, H3-10 were individual Hiseq 2500 single-end 100 nucleotide runs.

Std. AGO CLIP samples	RNAse	HSPC 117	T4 RNA ligase	complex MW	mapped CLIP reads	unique non-chimeric reads	unique miR-last chimeras	unique miR-first chimeras	chim. fraction	index	seq run
std-CLIP1	A, low, pre	NA	+	130-160	3,938,693	58,027	96	31	0.002	N/A	H4
std-CLIP2	A, low, pre	NA	+	130-160	627,156	79,894	47	200	0.003	N/A	H5
std-CLIP3	A, low, pre	NA	+	130-160	1,006,204	158,404	408	3963	0.028	N/A	H6
std-CLIP4	A, low, pre	NA	+	130-160	2,171,008	181,062	379	1864	0.012	N/A	H7
std-CLIP5	A, low, pre	NA	+	130-160	360,494	109,251	264	1596	0.017	CTCC	H8
std-CLIP6	A, low, pre	NA	+	130-160	720,147	340,404	107	716	0.002	GATC	H8
std-CLIP7	A, low, pre	NA	+	130-160	727,251	337,509	143	672	0.002	GCAT	H8
Mock1	A, low, pre	NA	+	130-160	1,183,802	558,876	555	6797	0.004	GTGC	H9
Mock2	A, low, pre	NA	+	130-160	1,443,223	632,342				GTGC	H10
Mock3	A, low, pre	NA	+	130-160	626,822	361,971				ACCG	H9
Mock4	A, low, pre	NA	+	130-160	629,102	339,542				ACCG	H10
LNA122-1	A, low, pre	NA	+	130-160	987,907	477,442	1004	12710	0.005	CATG	H9
LNA122-2	A, low, pre	NA	+	130-160	4,166,356	1,216,062				CATG	H10
LNA122-3	A, low, pre	NA	+	130-160	732,117	406,191				TGCA	H9
LNA122-4	A, low, pre	NA	+	130-160	605,410	414,305				TGCA	H10
Miravirsen-1	A, low, pre	NA	+	130-160	2,411,254	680,337	899	8084	0.004	TCAC	H9
Miravirsen-2	A, low, pre	NA	+	130-160	1,654,982	741,674				TCAC	H10
Miravirsen-3	A, low, pre	NA	+	130-160	876,350	284,679				AGTG	H9
Miravirsen-4	A, low, pre	NA	+	130-160	793,553	313,798				AGTG	H10
**CLEAR-CLIP Samples**	**RNAse**	**HSPC 117**	**T4 RNA ligase**	**complex MW**	**mapped CLIP reads**	**unique CLIP reads**	**unique miR-last chimeras**	**unique miR-first chimeras**	**chim. fraction**	**index**	**seq run**
1	A, low, pre	-	-	130-160	680,957	326,728	68	431	0.002	TCAC	H3
2	A, low, pre	-	-	130-160	212,575	100,860	36	109	0.001	AGTG	H3
3	A, low, pre	+	-	130-160	46,205	18,790	13	54	0.004	TACG	H3
4	A, low, pre	+	-	130-160	515,053	71,727	128	155	0.004	ATGC	H3
5	A, low, pre	+	+	130-160	204,482	45,824	228	847	0.023	CCGA	H3
6	A, low, pre	+	+	130-160	140,680	35,085	55	419	0.014	CTCC	H3
7	A, low, pre	++	-	130-160	292,522	64,007	165	85	0.004	GGCT	H3
8	A, low, pre	++	-	130-160	231,841	53,924	58	58	0.002	GAGG	H3
9	A, high, pre	++	-	130-160	688,170	126,717	107	92	0.002	AACC	H3
10	A, high, pre	++	-	130-160	370,362	89,241	69	62	0.001	ACTC	H3
11	T1, pre	++	-	130-160	464,863	101,169	39	78	0.001	TTGG	H3
12	T1, pre	++	-	130-160	325,086	83,510	31	52	0.001	TGAG	H3

Supplementary Table 5. Enrichments of interaction classes defined by k-means clustering analysis of Huh-7.5 miRNA-target interactions. Enrichment values for miRNAs with >50 identified interactions in seven interaction classes defined in Huh7.5 cells. Values are –log(p-value), calculated by Fisher's exact test. Positive values shaded in red indicate overrepresentation in a given class (enrichment), and negative values shaded in blue indicate underrepresentation (depletion).

	k group						
**microRNA**	**1**	**2**	**3**	**4**	**5A**	**5B**	**6**
hsa-let-7a-5p	4.49	0.81	-1.13	-3.38	0.07	-0.93	0.00
hsa-miR-101-3p	4.47	1.87	-0.92	0.07	0.34	-3.51	-3.16
hsa-miR-103a-3p	-2.70	20.12	-0.30	-2.96	-7.29	-3.54	2.00
hsa-miR-106b-5p	7.38	20.94	-3.35	-1.06	-6.55	-5.92	-3.38
hsa-miR-122-3p	18.69	0.42	-2.64	-0.97	-1.97	-1.82	-2.36
hsa-miR-122-5p	0.47	34.18	22.18	-34.02	-20.66	-9.66	-3.29
hsa-miR-125a-5p	0.48	-1.59	-0.16	-0.82	1.22	0.72	0.20
hsa-miR-1269a	0.42	-0.25	-0.98	-1.20	-2.34	-0.28	6.72
hsa-miR-130a-3p	-15.66	-0.28	2.31	-15.46	84.34	-10.47	-5.20
hsa-miR-130b-3p	-1.46	4.14	-0.31	-1.77	2.82	-0.74	-0.53
hsa-miR-140-3p	2.30	0.94	0.00	0.28	-1.07	-1.25	-0.74
hsa-miR-146a-5p	-3.35	-1.73	0.10	0.05	2.66	2.17	-0.13
hsa-miR-148a-3p	4.22	-0.06	-1.24	-1.37	-0.82	1.17	-0.28
hsa-miR-151a-3p	0.16	3.46	-4.13	-1.34	-0.84	2.96	-0.59
hsa-miR-151a-5p	0.22	0.77	-0.21	0.69	-1.22	-0.30	0.00
hsa-miR-15a-5p	-1.01	-0.48	15.63	-1.82	-6.79	0.32	-0.31
hsa-miR-15b-5p	15.74	2.89	0.38	-2.17	-8.37	-1.10	-5.83
hsa-miR-16-5p	-7.72	-30.14	12.79	-3.34	93.49	-8.67	-5.25
hsa-miR-17-3p	0.06	2.96	-1.82	-2.57	-1.22	1.37	0.05
hsa-miR-17-5p	-31.45	-22.46	21.48	0.78	-20.53	31.46	2.88
hsa-miR-181a-5p	-6.41	-3.28	-2.14	6.14	-2.33	13.64	-0.11
hsa-miR-181b-5p	-12.06	-9.17	-2.89	3.67	1.85	1.36	7.22
hsa-miR-182-5p	0.70	0.90	-4.85	-0.96	4.29	-0.93	0.00
hsa-miR-185-5p	4.49	-0.38	-5.45	-3.40	-0.06	5.07	-0.72
hsa-miR-186-5p	-43.74	-29.11	24.04	17.54	15.50	-0.23	-6.51
hsa-miR-188-5p	-0.40	0.00	-1.24	4.67	-0.78	-0.08	0.00
hsa-miR-18a-5p	0.24	10.58	2.19	-9.38	-9.37	0.04	-0.46
hsa-miR-191-5p	-2.66	-5.67	8.60	0.96	7.11	-0.04	-7.85
hsa-miR-192-5p	0.46	3.76	0.41	-0.15	-3.39	-0.73	-0.56
hsa-miR-193a-3p	2.68	-0.88	6.91	-1.45	-0.70	-1.39	-1.13
hsa-miR-193b-3p	-0.20	-0.46	6.29	-1.64	0.35	-3.46	0.43
hsa-miR-194-5p	-11.60	-13.92	0.00	15.85	-0.39	22.11	-3.29
hsa-miR-196a-5p	-8.58	-5.47	1.05	-1.43	0.87	3.29	4.35
hsa-miR-197-3p	0.33	0.36	0.32	-0.07	0.07	-0.22	-0.53
hsa-miR-19a-3p	67.56	-50.35	-0.93	-4.14	-2.23	-3.80	5.46
hsa-miR-19b-3p	9.23	-6.19	0.19	-0.15	-1.52	-1.06	0.79
hsa-miR-20a-5p	-12.70	-3.53	11.45	-0.65	-3.90	3.78	2.01

hsa-miR-21-5p	-1.36	0.92	-6.02	-2.96	-0.21	1.83	5.53
hsa-miR-210	-3.12	0.23	0.12	-0.29	0.30	-1.02	2.85
hsa-miR-22-3p	2.73	0.00	-0.63	-1.74	0.18	0.15	-0.21
hsa-miR-221-3p	-1.39	2.56	-0.74	-0.07	0.07	-1.82	1.33
hsa-miR-222-3p	-3.69	0.51	-5.65	-0.67	4.79	0.21	1.31
hsa-miR-23a-3p	71.18	0.93	-29.37	-0.48	-9.90	1.46	-14.59
hsa-miR-24-3p	5.39	2.88	-2.62	-3.81	-0.94	0.25	-0.99
hsa-miR-25-3p	-2.45	3.98	-1.71	-1.76	4.77	0.12	-1.06
hsa-miR-26a-5p	-2.51	-13.79	-1.15	1.09	-1.31	18.11	2.03
hsa-miR-26b-5p	0.23	-0.46	-3.07	0.00	-3.29	5.61	0.80
hsa-miR-27a-3p	15.95	29.77	-5.75	-0.14	0.32	-40.22	-10.49
hsa-miR-28-5p	3.52	-0.30	-1.10	-1.91	0.08	0.24	0.06
hsa-miR-29a-3p	1.97	0.30	-0.34	-0.46	4.78	-0.74	-4.81
hsa-miR-30a-5p	-3.67	1.28	4.47	29.36	-1.70	-5.43	-15.66
hsa-miR-30b-5p	-0.94	0.00	0.40	2.88	0.72	-2.31	-0.38
hsa-miR-30c-5p	0.02	-12.86	0.35	18.76	0.08	-1.50	-0.28
hsa-miR-32-5p	0.15	-2.45	0.34	-0.23	1.41	-0.20	0.62
hsa-miR-320a	-52.76	44.53	-28.47	3.46	-2.32	-13.76	20.24
hsa-miR-320c	-1.10	3.30	-2.45	-0.51	-1.22	-0.58	3.03
hsa-miR-33a-5p	3.25	4.54	-5.06	-1.26	0.00	-1.37	-0.26
hsa-miR-365a-3p	8.83	0.15	4.40	-2.52	-0.27	-4.49	-5.46
hsa-miR-374a-5p	7.41	-10.98	-0.04	6.85	-6.26	8.85	-8.20
hsa-miR-378a-3p	3.52	0.63	-3.44	-1.21	0.08	-1.87	0.72
hsa-miR-421	-2.27	-1.21	1.27	1.41	2.88	-0.15	-1.15
hsa-miR-423-3p	0.17	0.55	-1.52	-0.95	-1.11	1.31	0.77
hsa-miR-423-5p	1.73	3.39	-1.67	-2.41	-0.15	-0.64	-0.05
hsa-miR-425-5p	-0.41	0.80	0.95	-0.36	-4.06	1.01	0.00
hsa-miR-4279	1.14	-0.58	-6.80	-2.59	11.86	-0.86	0.08
hsa-miR-4286	1.57	-1.89	-3.25	0.86	2.11	0.00	-0.21
hsa-miR-4455	5.95	-0.26	-1.81	-1.36	3.29	-0.50	-2.08
hsa-miR-4488	5.59	-6.87	-3.03	-0.23	-0.05	-3.49	7.92
hsa-miR-454-3p	-4.58	-16.41	-1.76	-1.29	0.78	21.60	1.69
hsa-miR-455-3p	49.71	-1.02	-9.27	-0.32	0.00	-5.60	-10.68
hsa-miR-483-3p	3.38	1.85	-3.83	-0.52	-1.49	-0.36	0.17
hsa-miR-483-5p	-1.24	-0.39	-0.07	-0.07	3.11	-2.02	1.62
hsa-miR-484	3.04	5.35	-2.35	-1.30	-2.05	-1.12	-0.14
hsa-miR-500a-5p	-0.80	0.31	1.05	-0.73	0.00	-1.82	1.59
hsa-miR-5684	13.74	-1.38	-2.94	2.63	-0.74	-1.82	-2.84
hsa-miR-574-3p	1.09	1.02	-1.81	0.00	0.00	-2.13	0.61
hsa-miR-574-5p	-1.15	0.05	-6.66	0.03	-0.29	-1.29	11.84
hsa-miR-671-5p	-1.69	-1.73	-1.86	-0.26	-1.90	1.38	8.25
hsa-miR-885-5p	-0.79	0.57	-0.21	0.54	-2.59	0.33	0.86
hsa-miR-92a-3p	-3.56	13.48	-11.21	-0.08	0.38	-1.07	1.17
hsa-miR-92b-3p	0.13	5.57	-1.52	-0.07	-0.33	-0.06	-1.18
hsa-miR-93-5p	-11.68	-1.67	7.20	-0.07	-0.25	-4.78	9.97

hsa-miR-96-5p	1.73	-2.03	-1.70	0.00	0.00	1.23	0.38

Supplementary Table 6. 3'-UTR regions used in single-cell flow cytometry assays.

Figure panel	microRNA	Site type	MFE (kcal/mol), paralog 1	MFE (kcal/mol) paralog 2	gene	chr	start	end	strand
Supp. 10b	miR-30a	perfect	-38.8	-19.2	NA	NA	NA	NA	NA
Supp. 10b	miR-30c	perfect	-39.7	-21.9	NA	NA	NA	NA	NA
Supp. 10b	miR-125a	perfect	-46.8	-38.2	NA	NA	NA	NA	NA
Supp. 10b	miR-125b	perfect	-42.6	-34.4	NA	NA	NA	NA	NA
8b (and 8j)	miR-30a/c	8mer	-27.4 (miR-30c)	-22.5 (miR-30a)	Y-RNA	6	47731627	47731691	+
8d	miR-30a	Non-canonical, seedless	-21.3	-12.6	Pgrmc1	X	34144913	34144987	+
8e	miR-30a	8mer mismatch, 3' pairing	-23.5	-10.9	Naa15	3	51278463	51278537	+
8f	miR-30c	6mer, 3' pairing	-19.7	-16.9	Kpna1	16	36035854	36036009	+
8g	miR-30c	7mer-m8, 3' pairing	-21.6	-15.9	Ahnak	19	9093118	9093212	+
8h	miR-30c	bulged 7mer-m8, 3' pairing	-17.8	-11.7	Lin28b	10	45099044	45099108	-
8i	miR-30c	8mer mismatch (G-U wobble), 3' pairing	-25.6	-16.6	Ctgf	10	24318351	24318510	+
8k	miR-125a	bulged 7mer-m8, 3' pairing	-29.4	-21.5	Ap1s1	5	137511175	137511239	-
8l	miR-125a	7merm8, 3' pairing	-26.4	-22.7	Sox11	12	28020092	28020252	-
8m	miR-125a	Offset 6mer, 3' pairing	-25.6	-23.7	Ttbx2	2	120562261	120562414	-

Supplementary Table 7. Primers used in this study.

Primer	Sequence (5' to 3')	use
preA-L32	/5rApp/GTGTCAGTCACTTCCAGCGG/3ddC	Pre-adenylated 3' linker for CLIP
RL5D	OH-rArGrGrGrArGrGrArCrGrArUrGrCrGrGrNrNrNrNrG-OH	5' RNA linker for CLIP
P3	CCGCTGGAAGTGACTGACAC	RT and RT-PCR in CLIP protocol
P5	AGGGAGGACGATGCGG	RT-PCR in CLIP protocol
Index 1-CTAG	AATGATACGGCGACCACCGAGATCTACACTCTTTCCCTACACGACGCTCTTCCGATCTCTAGAGGGAGGACGATGCGG	2nd CLIP PCR 5'/ indexing
Index 2-GATC	AATGATACGGCGACCACCGAGATCTACACTCTTTCCCTACACGACGCTCTTCCGATCTGATCAGGGAGGACGATGCGG	2nd CLIP PCR 5'/ indexing
Index 3-CGTA	AATGATACGGCGACCACCGAGATCTACACTCTTTCCCTACACGACGCTCTTCCGATCTCGTAAGGGAGGACGATGCGG	2nd CLIP PCR 5'/ indexing
Index 4-GCAT	AATGATACGGCGACCACCGAGATCTACACTCTTTCCCTACACGACGCTCTTCCGATCTGCATAGGGAGGACGATGCGG	2nd CLIP PCR 5'/ indexing
Index 5-GTGC	AATGATACGGCGACCACCGAGATCTACACTCTTTCCCTACACGACGCTCTTCCGATCTGTGCAGGGAGGACGATGCGG	2nd CLIP PCR 5'/ indexing
Index 6-ACCG	AATGATACGGCGACCACCGAGATCTACACTCTTTCCCTACACGACGCTCTTCCGATCTACCGAGGGAGGACGATGCGG	2nd CLIP PCR 5'/ indexing
Index 7-CATG	AATGATACGGCGACCACCGAGATCTACACTCTTTCCCTACACGACGCTCTTCCGATCTCATGAGGGAGGACGATGCGG	2nd CLIP PCR 5'/ indexing
Index 8-TGCA	AATGATACGGCGACCACCGAGATCTACACTCTTTCCCTACACGACGCTCTTCCGATCTTGCAAGGGAGGACGATGCGG	2nd CLIP PCR 5'/ indexing
Index 9-TCAC	AATGATACGGCGACCACCGAGATCTACACTCTTTCCCTACACGACGCTCTTCCGATCTTCACAGGGAGGACGATGCGG	2nd CLIP PCR 5'/ indexing
Index 10-AGTG	AATGATACGGCGACCACCGAGATCTACACTCTTTCCCTACACGACGCTCTTCCGATCTAGTGAGGGAGGACGATGCGG	2nd CLIP PCR 5'/ indexing
Index 11-TACG	AATGATACGGCGACCACCGAGATCTACACTCTTTCCCTACACGACGCTCTTCCGATCTTACGAGGGAGGACGATGCGG	2nd CLIP PCR 5'/ indexing
Index 12-ATGC	AATGATACGGCGACCACCGAGATCTACACTCTTTCCCTACACGACGCTCTTCCGATCTATGCAGGGAGGACGATGCGG	2nd CLIP PCR 5'/ indexing
Index 13-CCGA	AATGATACGGCGACCACCGAGATCTACACTCTTTCCCTACACGACGCTCTTCCGATCTCCGAAGGGAGGACGATGCGG	2nd CLIP PCR 5'/ indexing
Index 14-CTCC	AATGATACGGCGACCACCGAGATCTACACTCTTTCCCTACACGACGCTCTTCCGATCTCTCCAGGGAGGACGATGCGG	2nd CLIP PCR 5'/ indexing
Index 15-GGCT	AATGATACGGCGACCACCGAGATCTACACTCTTTCCCTACACGACGCTCTTCCGATCTGGCTAGGGAGGACGATGCGG	2nd CLIP PCR 5'/ indexing

Index 16-GAGG	AATGATACGGCGACCACCGAGATCTACACTCTTTCCCTACACGACGCTCTTCCGATCTGAGGAGGGAGGACGATGCGG	2nd CLIP PCR 5'/ indexing
Index 17-AACC	AATGATACGGCGACCACCGAGATCTACACTCTTTCCCTACACGACGCTCTTCCGATCTAACCAGGGAGGACGATGCGG	2nd CLIP PCR 5'/ indexing
Index 18-ACTC	AATGATACGGCGACCACCGAGATCTACACTCTTTCCCTACACGACGCTCTTCCGATCTACTCAGGGAGGACGATGCGG	2nd CLIP PCR 5'/ indexing
Index 19-TTGG	AATGATACGGCGACCACCGAGATCTACACTCTTTCCCTACACGACGCTCTTCCGATCTTTGGAGGGAGGACGATGCGG	2nd CLIP PCR 5'/ indexing
Index 20-TGAG	AATGATACGGCGACCACCGAGATCTACACTCTTTCCCTACACGACGCTCTTCCGATCTTGAGAGGGAGGACGATGCGG	2nd CLIP PCR 5'/ indexing
MSFP3	CAAGCAGAAGACGGCATACGAGATCCGCTGGAAGTGACTGACAC	2nd CLIP PCR, 3'
mm_Gnaq_f	TCAAAAGCAGACACCTTCTCC	qRT-PCR
mm_Gnaq_r	CACGCTCAAGATCCCATACA	qRT-PCR
mm_Map6d1_f	CTCTGGCTGTTCTTGCCTTT	qRT-PCR
mm_Map6d1_r	ACGCAGCTGTAGTAGCGACC	qRT-PCR
mm_NBEA_f	aggagttcagccatgcatct	qRT-PCR
mm_NBEA_r	tggaaacttcagcaccatga	qRT-PCR
mm_Gosr2_f	AGACTGCTTGTCTGCCCTCT	qRT-PCR
mm_Gosr2_r	GTCATGGAGCCCCTATACCA	qRT-PCR
mm_Ctnnd1_f	GCACCCTTGATgcAGAAGAT	qRT-PCR
mm_Ctnnd1_r	GTCCAGCGAAGAAAGGAAAA	qRT-PCR
mm_Serinc3_f	CCACTACAGAGGCACGGG	qRT-PCR
mm_Serinc3_r	TCAGCAGTTAGGATCCGCTC	qRT-PCR
mm_Rfx7_f	GCTGTCCCTAGCCCCATT	qRT-PCR
mm_Rfx7_f	acttgcacattgaggggaag	qRT-PCR
mm_Comt_f	AGCCTAGGTGTCGCAGGAG	qRT-PCR
mm_Comt_r	CTCGGGGTCAGACCTGTG	qRT-PCR
mm_Glul_f	TTGCTTGATGCCTTTGTTCA	qRT-PCR
mm_Glul_r	CTCCTGACCTGTTCACCCAT	qRT-PCR
mm_Chchd10_f	TGATCTCATAGGAGCAGGGC	qRT-PCR
mm_Chchd10_r	TTCAGTGGGGGAAATTCAGA	qRT-PCR
mm_SMAD7_f	CTTCTCCTCCCAGTATGCCA	qRT-PCR
mm_SMAD7_r	GAACGAATTATCTGGCCCCT	qRT-PCR
mm_Chd9_f	AATTGCCAAGGCTACCACAG	qRT-PCR
mm_Chd9_r	GGCCTGAAGAGCTGTGAGAT	qRT-PCR
mm_Rpl10a_f_1 (Fig. 4e)	CAGGATACGTGGGATCTGCT	qRT-PCR
mm_Rpl10a_r_2 (Fig. 4e)	AGGCGCTCAAGAAGCTTAAC	qRT-PCR
mm_Gapdh_f	GACCTCATGGCCTACATGG	qRT-PCR
mm_Gapdh_r	TCTTGCTCAGTGTCCTTGC	qRT-PCR
mm_Rpl10a_f2 (Supp Fig. 2b)	GACCCTCAGAAGGACAAACG	qRT-PCR
mm_Rpl10a_r2 (Supp Fig. 2b)	AGAACGCACACCGAGAAC	qRT-PCR
ec_16S_f	CAGCCACACTGGAACTGAGA	qRT-PCR
ec_16S_r	GTGCAATATTCCCCACTGCT	qRT-PCR
ec_gada_f	GGTGATGCGCATTATGTGTC	qRT-PCR
ec_gada_r	CTTCCAGCAACAGTTCAGCA	qRT-PCR
ec_dnak_f	TCGTATGCCAATGGTTCAGA	qRT-PCR
ec_dnak_r	TAACGTCTTTACGCGGCTCT	qRT-PCR
ec_groes_f	ACGGTGTGAAATCCGAGAAG	qRT-PCR

Ec_groes_r	AATTGCCAGAATGTCGCTTT	qRT-PCR
125A-NP	TCACAGGTTAAAGGGTCTCAGGGA	Northern
125B-NP	TCACAAGTTAGGGTCTCAGGGA	Northern
30A-NP	CTTCCAGTCGAGGATGTTTACA	Northern
30C-NP	GCTGAGAGTGTAGGATGTTTACA	Northern

Supplementary References

1. Chi, S. W., Zang, J. B., Mele, A. & Darnell, R. B. Argonaute HITS-CLIP decodes microRNA-mRNA interaction maps. *Nature* **460**, 479-486 (2009).
2. Moore, M. J. et al. Mapping Argonaute and conventional RNA-binding protein interactions with RNA at single-nucleotide resolution using HITS-CLIP and CIMS analysis. *Nat Protoc* **9**, 263-293 (2014).
3. He, M. et al. Cell-type-based analysis of microRNA profiles in the mouse brain. *Neuron* **73**, 35-48 (2012).
4. Cheng, L. C., Pastrana, E., Tavazoie, M. & Doetsch, F. miR-124 regulates adult neurogenesis in the subventricular zone stem cell niche. *Nat Neurosci* **12**, 399-408 (2009).
5. Kole, A. J., Swahari, V., Hammond, S. M. & Deshmukh, M. miR-29b is activated during neuronal maturation and targets BH3-only genes to restrict apoptosis. *Genes Dev* **25**, 125-130 (2011).
6. Clovis, Y. M., Enard, W., Marinaro, F., Huttner, W. B. & De Pietri Tonelli, D. Convergent repression of Foxp2 3'UTR by miR-9 and miR-132 in embryonic mouse neocortex: implications for radial migration of neurons. *Development* **139**, 3332-3342 (2012).
7. Kanehisa, M. et al. Data, information, knowledge and principle: back to metabolism in KEGG. *Nucleic Acids Res* **42**, D199-205 (2014).
8. Kanehisa, M. & Goto, S. KEGG: kyoto encyclopedia of genes and genomes. *Nucleic Acids Res* **28**, 27-30 (2000).
9. Shi, Z. et al. MiR-124 governs glioma growth and angiogenesis and enhances chemosensitivity by targeting R-Ras and N-Ras. *Neuro Oncol* **16**, 1341-1353 (2014).
10. Tan, X. et al. The CREB-miR-9 negative feedback minicircuitry coordinates the migration and proliferation of glioma cells. *PLoS One* **7**, e49570 (2012).
11. Huse, J. T. et al. The PTEN-regulating microRNA miR-26a is amplified in high-grade glioma and facilitates gliomagenesis in vivo. *Genes Dev* **23**, 1327-1337 (2009).
